# A select inhibitor of MORC2 encapsulated by chimeric membranecoated DNA nanocage target alleviation TNBC progression

**DOI:** 10.1016/j.mtbio.2025.101497

**Published:** 2025-01-19

**Authors:** Xiaohan Su, Yunbo Luo, Yali Wang, Peng Qu, Jun Liu, Shiqi Han, Cui Ma, Shishan Deng, Qi Liang, Xiaowei Qi, Panke Cheng, Lingmi Hou

**Affiliations:** a Breast Surgery, Sichuan Cancer Hospital & Institute, Sichuan Cancer Center, School of Medicine, University of Electronic Science and Technology of China, Chengdu, China; b Department of Breast Surgery, Mianyang 404 hospital, Mianyang, China; c Department of Breast and Thyroid Surgery, Biological Targeting Laboratory of Breast Cancer, Academician (expert) Workstation, Affiliated Hospital of North Sichuan Medical College, Nanchong, China; d Department of Laboratory Medicine, Affiliated Hospital of North Sichuan Medical College, Nanchong, China; e Institute of Cardiovascular Diseases & Department of Cardiology, Sichuan Provincial People's Hospital, School of Medicine, University of Electronic Science and Technology of China, Chengdu, China; f Department of Mathematics, Army Medical University, Chongqing, China; g Department of Breast Surgery, Southwest Hospital, Army Medical University, Chongqing, China; h Ultrasound in Cardiac Electrophysiology and Biomechanics Key Laboratory of Sichuan Province, Chengdu, China

**Keywords:** MORC2, Angoline, Biomimetic nanodrug delivery system, Triple-negative breast cancer

## Abstract

Triple-negative breast cancer (TNBC) is the most malignant type of breast cancer and lacks effective targeted therapeutic drugs, resulting in a high recurrence rate and worse outcome. In this study, bioinformatic analysis and a series of experiments demonstrated that MOCR2 was highly expressed in TNBC and closely associated with poor prognosis, indicating that MOCR2 may be a potential therapeutic target for TNBC. Subsequently, Angoline was identified as an inhibitor of MORC2 protein by high-throughput screening and can significantly kill the TNBC cells by blocking cell cycle and inducing apoptosis. Furthermore, the biomimetic nanodrug delivery system (PMD) was designed by encapsulating tetrahedral DNA nanostructures with biomimetic cell membrane, and it can efficiently evade the phagocytosis of immune system and target TNBC tissue. Additionally, PMD can markedly enhance the killing effect of Angoline on TNBC tumors. Therefore, PMD-enveloped Angoline provide a highly effective targeted therapeutic regimen for TNBC and may improve the outcome for patients with TNBC.

## Graphical abstract

The hybrid membranes (TNMm) were derived from the membranes of TNBC cells, neutrophils and monocytes. Angoline was encapsulated in DNA tetrahedron (tDNAn) formed by annealing four ssDNAs. Subsequently, PMD was acquired by wrapping tDNAn with TNMm and attaching EGF_Peptides to TNMm surface by DSPE. PMD transports though blood circulation and avoids phagocytosis by neutrophils and monocytes, ultimately targeting TNBC tissue due to over-expressed EGFR in TNBC cells. Angoline is released after PMD enters TNBC cells and binds to MORC2 protein, causing TNBC cells death through cell cycle arrest and apoptosis pathway. This graphic was created with BioRender.com.

**Figure:**
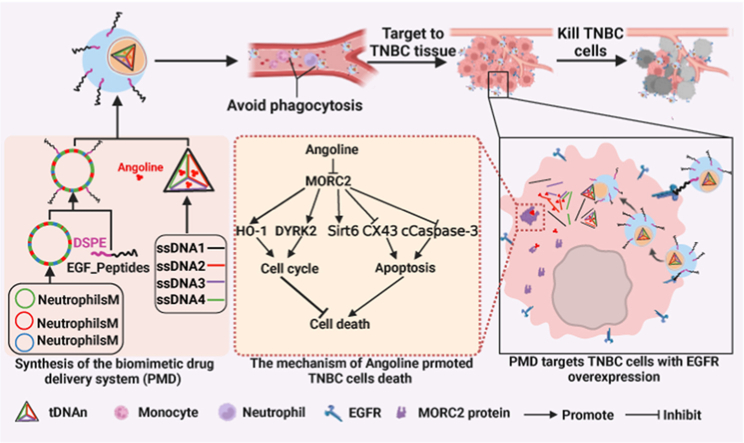

## 1 Introduction

Breast cancer (BC) is the most common malignant tumor among women and poses a serious threat to women's life and physical health [1]. Classically, BC can be divided into four subtypes according to the expression of estrogen receptor (ER), progesterone receptor (PR) and human epidermal growth factor receptor 2 (HER2). Among the four subtypes, triple-negative breast cancer (TNBC) comprises 10–15 % of cases and exhibits the worst outcome [2], which is mainly attributed to the poor biological behavior of TNBC and lacking effective therapeutic targets. Over the past few decades, medical scientists have focused on finding key therapeutic targets for BC, but few targeted drugs can benefit patients with TNBC. Although PARP enzyme inhibitors are effective for patients with BRCA germline mutations [3], fewer patients can benefit from such regime because of 9.3–15.4 % patients with TNBC presenting BRCA germline mutations [4]. Besides, immunotherapy has demonstrated promising efficacy in various malignant tumors, such as melanoma [5], non-small-cell lung cancer [6], and renal-cell carcinoma [7]. While, some of patients with TNBC can only benefit from immunotherapy in combination with other regimes, such chemotherapy, radiotherapy, which will increase the side effects for patients. Therefore, finding the effective targets is the most urgent challenge to improve the therapeutic effect for TNBC.

Previous studies have demonstrated that cells can die in a variety of ways, including cell cycle arrest, apoptosis, autophagic cell death, ferroptosis, pyroptosis and cuproptosis [8]. Among them, cell cycle arrest typically occurs at an early phase and often culminates in irreversible cell death, as it is positioned upstream of multiple death-regulating pathways. This enables cell cycle arrest to act as a primary checkpoint, preemptively controlling the progression to subsequent stages that could trigger various cell death mechanisms [9]. Thus, finding the key cycle regulatory protein may provide a good therapeutic target for TNBC. Recently, some researches showed that Microrchidia CW-type zinc finger protein 2 (MORC2) protein can regulate cell cycle checkpoint activation during DNA damage through acetylation and affect DNA damage repair [10]. Additionally, it is overexpressed in a variety of tumors and promotes their progression [[11], [12], [13]]. MORC2 also has a dual role in estrogen-induced proliferation of breast cancer cells and resistance to anti-estrogenic therapy [14]. Moreover, high expression of MORC2 predicts poor response to neoadjuvant chemotherapy in TNBC [15], and another study showed that MORC2-mutant can promote the invasion and metastasis of TNBC [11]. Therefore, targeting MORC2 protein may provide an effective therapeutic strategy for TNBC.

Currently, many techniques can be used to target specific proteins or molecules, such as monoclonal antibody, RNA interference technique, CRISPR-Cas9 gene editing technology, small molecule compounds, and etc. Among them, small molecule compounds, referring to the natural compounds with molecular weight less than 900 Da, have shown outstanding strengths and broad application prospect in anti-tumor therapy. Many small molecule compounds have been successfully used in some malignant tumors, such as CDK4/6 inhibitors, alpelisib, and etc [[16], [17], [18], [19]]. The successful translational examples of small molecule inhibitors in anti-cancer therapy may be the following reasons: First, they can directly bind to the target proteins and inhibit the activity of the target proteins by substrate competition, altering the protein structure or hindering conformational changes. Then, small molecule inhibitors targeting specific proteins can be easily found by virtual screening and molecular docking [20], which also promoted the application of small molecule inhibitors in anti-tumor therapy. Moreover, the toxicity and pharmacokinetics of many small molecule drugs have been thoroughly studied, which enables small molecule inhibitors with anti-tumor effects to advance rapidly into Phase II clinical trials [21]. Therefore, small molecule inhibitors targeting specific proteins hold promise as anti-tumor therapies due to their high specificity and well-established development process. Nevertheless, the lack of targeting to tumor tissue may reduce its bioavailability.

Recent years, advances in nanotechnology and biomimetic cell membrane-camouflaged technology may further enhance the effects of small molecule inhibitors in anti-cancer therapy. The developed polyhedral DNA nanostructures have emerged as highly promising scaffolding materials for drug delivery systems. They exhibit outstanding biocompatibility, precise programmability, good reproducibility, structural predictability, and can enhance biostability through various modifications [22]. However, the stable persistence of DNA nanocages within the circulatory system remains challenging. First, DNA structures are easily degraded by nucleases, which reduces the stability and half-life of DNA nanostructures. Then, avoiding phagocytosis by the monocyte-macrophage system is another critical obstacle that must be addressed. Cancer cell membrane coating strategies have homologous targeting and immune-evasion capabilities [23]. However, the homologous targeting and immune-evasion capabilities of cancer cell membrane coating strategies are limited, and additional coating strategies are needed to further enhance these properties. Biomimetic cell membranes can be derived from a variety of cell membranes, including neutrophils membranes, monocytes membranes, tumor cell membranes, platelet membrane fragment and etc [24,25]. Biomimetic cell membrane-camouflaged DNA nanocages can protect the DNA from enzymatic degradation and extend its half-life. Moreover, hybrid-cell membranes can endow synthetic nanoparticles with a range of biological functions derived from the original source cells [26]. These biomimetic membranes mimic the surface properties of the source cell, making the nanoparticles (NPs) look like their own cells, thus evading recognition and phagocytosis by the immune system. Also, immunomodulatory molecules (such as CD47) carried on the surface of monocytes and neutrophil cell membranes can bind to receptors on immune cells, sending a "don't eat me" signal and inhibiting phagocytosis [27]. Actually, neutrophils, playing a key role in activating the immune cascade, recognize pathogen-associated molecular patterns (PAMPs) through surface receptors such as toll-like receptors (TLRs) and release inflammatory mediators and chemokines upon activation [28]. Thus, NPs camouflaged by neutrophils membranes can reduce the activation of neutrophils and reduce the immune cascade, which further allows the NPs to escape phagocytosis before reaching the tumor site. In addition to anti-phagocytosis, NPs camouflaged by cancer cell membranes are capable of homotypic targeting [29], which makes it easier for drugs to reach the tumor sites. In summary, hybrid-cell membranes can not only protect the DNA nanostructures from degradation, but also resist phagocytosis and promote the drug enriched in the tumor sites. Furthermore, due to the high expression of epidermal growth factor receptors (EGFR) on TNBC cell membranes, modifying epidermal growth factor-peptides (EGF_Peptides) is considered an effective strategy to improve the targeting efficiency of biomimetic nanodrug delivery system to the tumor sites. Therefore, leveraging nanotechnology and biomimetic cell membrane camouflage, small molecule inhibitors can be more efficiently delivered through the circulatory system to specifically target TNBC tissue.

In our study, we confirmed that MOCR2 is an excellent potential therapeutic target for TNBC by bioinformatics analysis and basic experiments. Subsequently, high-throughput screening and cytotoxicity tests confirmed that Angoline can target MOCR2 protein and inhibit the growth of TNBC cells by blocking cell cycle and inducing apoptosis. Furthermore, biomimetic nanodrug delivery system (PMD) was constructed to facilitate Angoline targeting to tumor sites. The results showed that the PMD is enriched in the tumor site and can significantly inhibit the growth of TNBC by delivering Angoline to the tumor sites. In conclusion, PMD-enveloped Angoline may be a promising targeted drug for TNBC.

## 2 Results and discussion

### 2.1 High expression of MOCR2 leads to worse biological behavior of TNBC

**Fig. 1:**
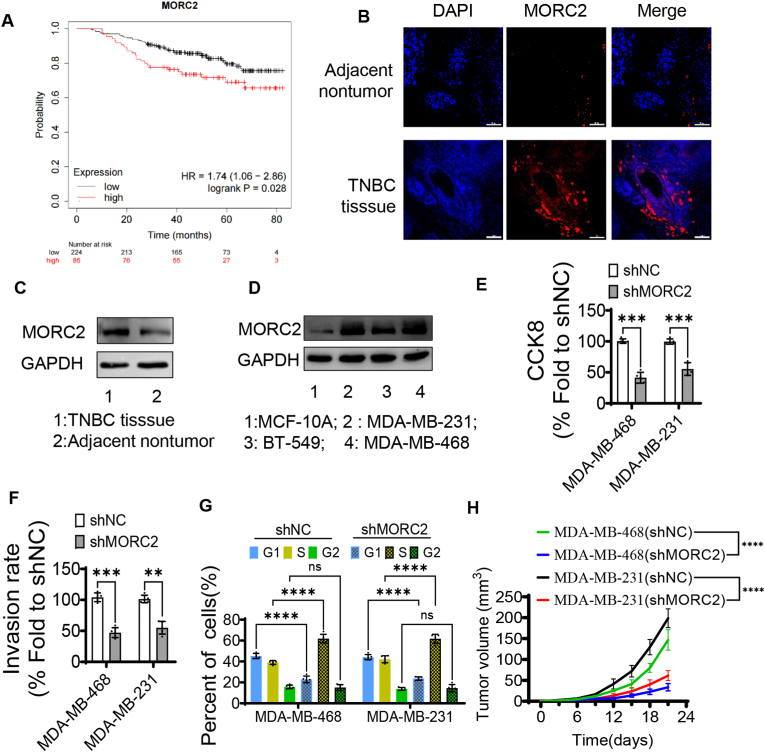
MORC2 is highly expressed in TNBC and is closely associated with poor biological behavior (high proliferation and invasion ability) and prognosis. (A) Kaplan-Meier survival curve for patients with high/low expression of MORC2. Data come from the KM-plotter database. (B) Immunofluorescence showed that the expression of MORC2 is significantly higher in TNBC tissue than adjacent nontumor tissue (scale = 100 μm). (C) Western blot results of MORC2 expression in TNBC tissue or adjacent nontumor tissue. (D) Western blot results of MORC2 expression in normal breast epithelial cell line (MCF-10A) or TNBC cell lines (MDA-MB-231, BT-549 and MDA-MB-468). (E) Knockdown of MORC2 decreased the proliferation capability of both MDA-MB-231 and BT-549. (F) Knockdown of MORC2 decreased the invasion capability of both MDA-MB-231 and BT-549. (G) Cell cycle changed after Knockdown of MORC2 in MDA-MB-231 and BT-549 cells. (H) Growth curves of inoculated xenograft tumors in shMORC2 or shNC groups. ∗∗P < 0.01; ∗∗∗P < 0.001; ∗∗∗∗P < 0.0001; ns, no significance.

MORC2, a chromatin remodeling enzyme, is highly expressed in various tumor, such as gastric cancer [30], colorectal cancer [12], and other cancers [31]. The bioinformatics analysis showed that MOCR2 is also overexpressed in breast cancer compared with normal breast tissue (Fig. S1A). Moreover, the expression of MOCR2 is significantly higher in TNBC than other molecular types (Fig. S1B). Meanwhile, high expression of MOCR2 exhibited worse overall survival (HR: 1.74, 95%CI: 1.06–2.86) compared with low expression in TNBC (Fig. 1A). Taken together, these results suggested that MOCR2 may promote the progression of TNBC. To verify the expression of MOCR2 in TNBC, we conducted a series of experiments by using the tissues from patients with TNBC. Immunofluorescence revealed that the expression of MOCR2 is significantly higher in tumor tissue than adjacent nontumor tissue (Fig. 1B). Also, immunohistochemistry showed that MOCR2 is highly expressed in TNBC tumor tissue (Fig. S2A). Then, western blot (WB) validated the higher expression of MOCR2 in TNBC (Fig. 1C). Moreover, MOCR2 was confirmed to be higher expression both in MDA-MB-231 and MDA-MB-468 cell lines, but lower expression in BT-549 cell line (Fig. 1D). Thus, MDA-MB-231 and MDA-MB-468 were used to conduct the following experiments.

In order to elucidate the mechanism that MOCR2 promote the progression of TNBC, we efficiently knockdown the expression of MOCR2 in two TNBC cell lines (MDA-MB-231 and MDA-MB-468) by lentivirus-mediated transfection of two shRNAs targeting MOCR2. The CCK8 assay showed that downregulation of MOCR2 significantly compromised the growth of both MDA-MB-231 and MDA-MB-468 cells 48 h after passage (Fig. 1E). Also, the invasion capacity was found to be significantly reduced in MOCR2 knockdown TNBC cells (Fig. 1F and Fig. S3A). Previous studies have shown that MORC2 plays an important role in the cell cycle, promoting cell proliferation by regulating the expression and function of multiple cell cycle regulatory proteins, such as p21, NAT10 and etc [32,33]. Therefore, we speculated that MOCR2 may promote TNBC proliferation by regulating the cell cycle. Flow cytometry was performed to detect cell cycle changes after knockdown of MOCR2 in TNBC cells. As we hypothesized, knockdown of MOCR2 dramatically compromised the transition from S phase to G2 phase, causing higher proportion of cells arrested in the S phase both in MDA-MB-231 and MDA-MB-468 cell lines (Fig. 1G and Fig. S4A). After validating the promotion of proliferation and invasion by MOCR2 in vitro, we further conducted a series of functional experiments in vivo to determine the effect of MOCR2 on tumor growth. We established TNBC xenograft models in BALB/c nude mice by inoculating TNBC cells stably transduced with MDA-MB-231 (shMORC2 or shNC) and MDA-MB-468 (shMORC2 or shNC), respectively. The tumor volumes of xenografts were monitored and recorded every 3 days. Three weeks after implantation, the mice were sacrificed and the xenografts were carefully harvested, weighed and photographed. The average volume and weight of xenografts were significantly reduced in the shMORC2 group compared to the shNC group (Fig. 1H, Fig. S5A, and Fig. S5B), indicating that MORC2 can promote tumor growth and may be a potential target for TNBC treatment. Therefore, inhibition of MOCR2 protein may be a promising treatment strategy for TNBC.

### 2.2 High-throughput screening of targeted-MOCR2 drugs and evaluation of efficacy in vitro

At present, many mature techniques can be used to target specific proteins, such as monoclonal antibody, RNA interference technique, CRISPR-Cas9 gene editing technology. While, most of these approaches are technically demanding and costly, which limits their widespread use in clinical settings and benefits only a select few patients. Fortunately, small molecule compounds can also target specific proteins and have many advantages compared with the above techniques. First, effective small molecule compounds targeting specific proteins can be easily found by the high-throughput screening and molecular interaction experiment in vitro. Then, new use of old drugs can get these small molecules into the clinic faster because of the well-known toxicity and pharmacokinetics from previously comprehensive studies [21]. Therefore, we searched for the potential small molecule inhibitors of MOCR2 protein by high-throughput screening.

**Fig. 2:**
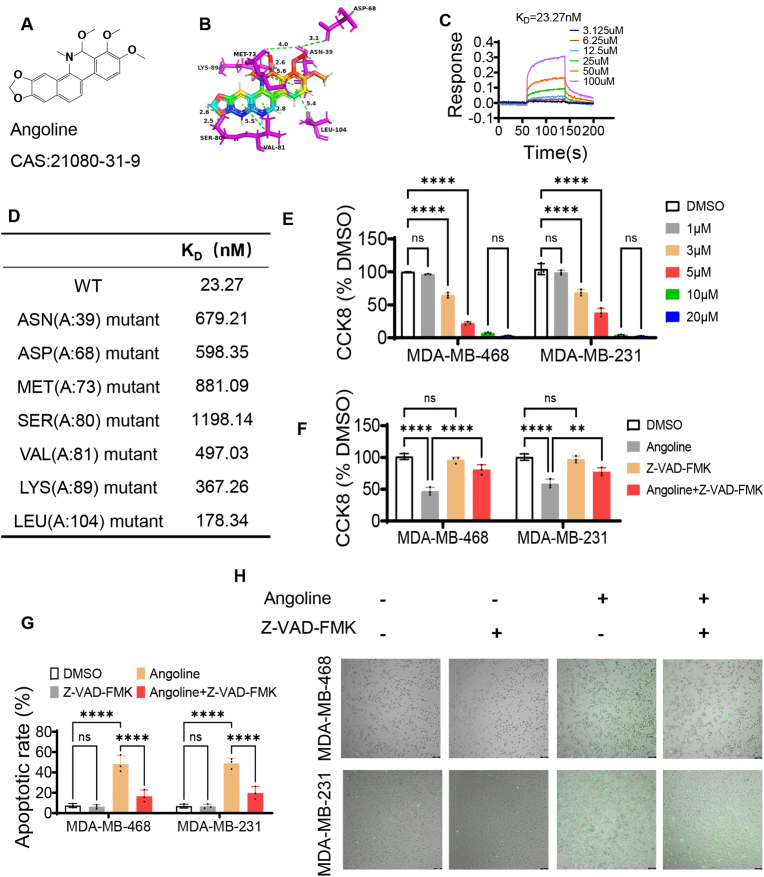
Angoline has strong binding capacity with MORC2 protein and causes TNBC cell death by inducing apoptosis. (A) Diagram of the chemical structure of Angoline. (B) Molecular docking software predicts binding sites, interaction forces and distances between Angoline and MORC2 protein. ASP, asparticacid; ASN, asparagine; MET, methionine; LYS, lysine; SER, serine; VAL, valine; LEU, leucine. (C) In vitro molecular interaction experiment showed that the binding strength between MORC2 protein and Angoline varies with the concentration of Angoline. (D) The binding force between MORC2 protein and Angoline increased with the mutation of amino acids at the binding site. (E) The killing effect of Angoline on TNBC cells was enhanced with increasing concentration. (F) The killing effect of Angoline on TNBC cells was reduced by apoptosis inhibitor (Z-VAD-FMK). (G) The apoptosis-inducing effect of Angoline on TNBC cells was weakened by apoptosis inhibitor (Z-VAD-FMK). (H) YO-PRO-1 staining showed that the pro-apoptotic effect of angoline on TNBC cells can be attenuated by apoptosis inhibitor (Z-VAD-FMK) ((bar, 100 μm)). ∗∗P < 0.01; ∗∗∗P < 0.001; ∗∗∗∗P < 0.0001; ns, no significance.

Topscience biological small molecule compound library was used to virtually screen out the potential small molecules by computer molecular docking. Subsequently, the top 10 compounds were validated on TNBC cells, and the results showed that Angoline had the strongest killing effect both on MAD-MB-231 and MAD-MB-468 cell lines (Figs. S6A–B). Then, a series of experiments were conducted to evaluate the binding ability between small molecule compounds and MORC2 protein. First, the optimal conditions for purification of MORC2 protein were investigated by univariate optimization method. Protein induction experiments were conducted under varying concentrations of IPTG (0 mM, 0.1 mM, 0.2 mM, 0.5 mM, 0.8 mM, 1 mM), temperature gradients (16 °C, 22 °C, 29 °C, 37 °C), and induction time (3 h, 6 h, 8 h, 10 h, 12 h). Finally, at 0.1 mM IPTG concentration, especially at 22 °C for 8 h, MORC2 protein expression reached the optimal level, which was verified by Coomassie blue staining (Figs. S7A–C). We cleared the other proteins by Binding/Wash Buffer 3 times and eluted the MORC2 protein with imidazole at 40 mM concentration to extract the high purity MORC2 protein (Fig. S7D). MORC2 protein with high purity was obtained according to the above conditions and verified by Coomassie blue staining (Fig. S7E). Then, surface plasmon resonance molecular interaction experiment was performed to detect the binding ability between small molecule compounds and MORC2 protein. The results showed that the K_D_ value between MORC2 protein and Angoline is the lowest, with 23.27 nM (Fig. S8A), which revealed a strong binding force between them. And the chemical structure of Angoline was shown in Fig. 2A. Moreover, molecular docking displayed that angoline can bind to multiple amino acids of MOCR2 protein through hydrogen bonds, Pi-Alkyl, Pi-Sigma, Pi-Alkyl bonds (Fig. 2B). Also, the binding strength between MORC2 and Angoline was dose-dependent on the concentration of Angoline (Fig. 2C). The K_D_ values increased significantly with the mutation of some amino acids in MORC2 protein, such as ASN, ASP, MET, SER, VAL, LYS and LEU (Fig. 2D). These dramatic changes further suggested that Angoline interacts with MORC2 protein via the amino acids mentioned above. In summary, Angoline may be a promising small molecule inhibitor for.

MORC2 protein and can be used to inhibit the growth of TNBC cells. Nevertheless, the optimal concentration of Angoline and the exact way of killing TNBC cells need to be further clarified.

### 2.3 Angoline kills TNBC cells by inducing cell cycle arrest and apoptosis pathway

CCK8 assay was performed to confirm the optimal killing concentration of Angoline on TNBC cells (MDA-MB-231, MDA-MB-468, and 4T1) in vitro. The inhibitory effect of Angoline on TNBC cell viability progressively increased with higher concentrations in both human and mouse breast cancer cell lines (Fig. 2E and Fig. S9A). These results suggested that the interaction between Angoline and MORC2 protein can induce the death of TNBC cells and the effect was concentration-dependent, which was further confirmed by trypan blue dye staining (Fig. S10A). Our previous results demonstrated that MORC2 promotes tumor cell proliferation by regulating the cell cycle, suggesting that targeting the MORC2 protein with Angoline may inhibit TNBC cells by inducing cell cycle arrest and promoting apoptosis. Thus, we further verified our speculation by detecting the cell viability and apoptosis rate of TNBC cells treated with Angoline and/or apoptosis inhibitor (Z-VAD-FMK). As expected, the toxic effect of Angoline on TNBC cells was reduced by Z-VAD-FMK (Fig. 2F). Then, the apoptosis rate significantly increased in TNBC cells treated with Angoline, but Z-VAD-FMK could significantly reduce the proapoptotic effect of Angoline on TNBC cells (Fig. 2G and Fig. S10B). Also, YO-PRO-1 staining confirmed that the proportion of apoptotic cells was reduced when Z-VAD-FMK was added in the Angoline treated group (Fig. 2H). Taken together, these results confirmed that Angoline inhibits TNBC cells through a pro-apoptotic pathway. While, the detailed pro-apoptotic mechanism needs to be further elucidated.

**Fig. 3:**
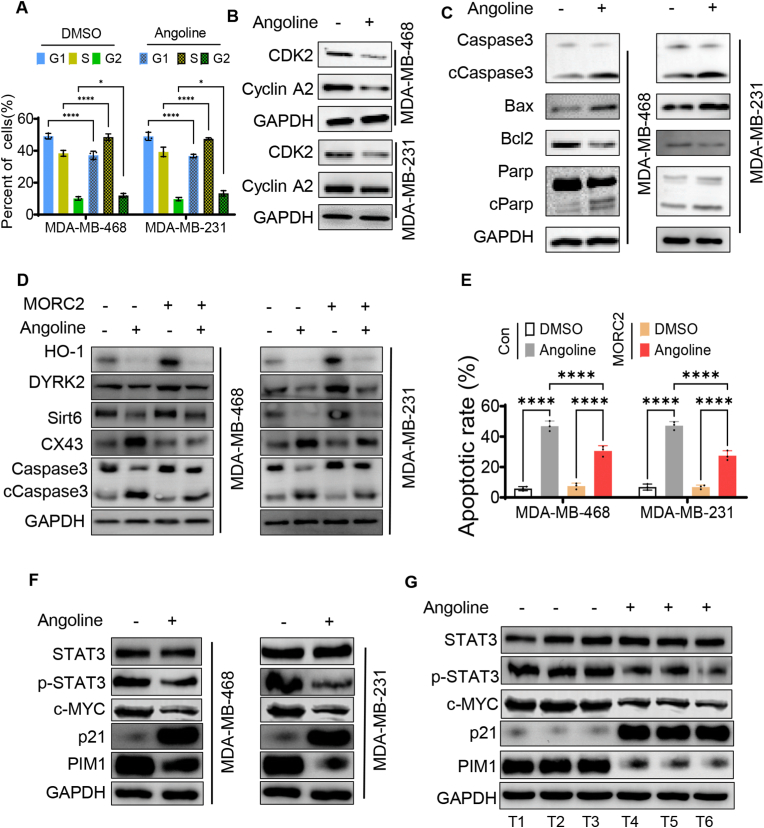
Angoline promotes TNBC cells apoptosis through inhibiting MORC2 protein and regulating cell cycle. (A) Cell cycle changes of TNBC cells treated with Angoline. (B) Changes of cell cycle-related protein expression in TNBC cells treated with Angoline. (C) Changes of apoptosis-related protein expression in TNBC cells treated with Angoline. (D) Changes of cell cycle and apoptosis-related protein expression in TNBC cells after MORC2 overexpressed and/or treated with Angoline. (E) Changes of apoptosis ratio in TNBC cells after MORC2 overexpressed and/or treated with Angoline. (F) Phosphotyrosine STAT3 (p-STAT3) was inhibited in TNBC cells treated with Angoline, leading to changes in the expression of proteins associated with cell cycle and apoptosis. (G) Phosphotyrosine STAT3 (p-STAT3) was inhibited in tumor-bearing mice treated with Angoline, leading to changes in the expression of proteins associated with cell cycle and apoptosis. ∗P < 0.05; ∗∗P < 0.01; ∗∗∗P < 0.001; ∗∗∗∗P < 0.0001; ns, no significance.

Previous studies demonstrated that cell cycle arrest can promote apoptosis by activating apoptosis-related proteins through a variety of signaling pathways and mechanisms [34]. In our study, higher proportion of TNBC cells remained in S and G2 phases after Angoline treatment (Fig. 3A and Fig. S11A), which revealed that Angoline may promote TNBC cell apoptosis by regulating the cell cycle. Thus, WB was performed to detect the changes of the cell cycle related proteins in TNBC cells treated with Angoline. The results showed that the expression of CDK2 and Cyclin A2 significantly decreased both in MDA-MB-231 and MDA-MB-468 cells after the treatment with Angoline (Fig. 3B). Additionally, the expression of Caspase-3 and PARP significantly decreased in the angoline group, but their active proteins (cleaved Caspase-3 and cleaved PARP) significantly increased. Moreover, Bax, as a key protein in the apoptotic cascade [35], was also seen increased in the angoline group. However, the apoptosis-suppressed protein, BCL-2, presented significantly lower expression in Angoline group than that in the control group. These molecular biology experiments demonstrated that Angoline exerts a significant pro-apoptotic effect by blocking the cell cycle of TNBC cells.

In order to further verify that Angoline promotes apoptosis of TNBC through interaction with MOCR2 protein, we constructed MORC2-overexpressed (MORC2-OV) TNBC cell lines and shMORC2 TNBC cell lines. Then, Angoline was applied to those TNBC cells and WB was performed to detect the changes of cell cycle-related and apoptosis-related proteins (Fig. 3D and Fig. S12A). HO-1 may regulate cell cycle progression by influencing the expression and activity of cell cycle regulatory proteins, such as Cyclin D1 and CDK4 [36]. DYRK2 is a key regulator of the cell cycle and plays an important role in the G1/S transition of the cell cycle [37]. Our study showed that the expression of HO-1 and DYRK2 significantly decreased in shMORC2 TNBC cells, which was consistent with TNBC cells treated with Angoline. On the contrary, the expression of HO-1 and DYRK2 both increased in MORC2-OV TNBC cells, which indicated that MORC2 may promote the cell cycle by upregulating the expression of HO-1 and DYRK2. Moreover, the declined levels of HO-1 and DYRK2 were hindereded in MORC2-OV TNBC cells treated with Angoline. These changes suggested that Angoline inhibits the function of MORC2 protein, resulting in the cell cycle arrest in TNBC cells. Apoptosis, autophagic cell death, and necrosis are the main pathways of cell death after the cell cycle arresting. In our study, previous results showed that knockdown of MORC2 can induce the apoptosis of TNBC cells. Therefore, we continued to detect the changes of proteins associated with apoptosis in TNBC cells treated with Angoline. CX43 and cleaved caspase-3 are pro-apoptosis-related proteins [38,39], both of which were seen increased in shMORC2 TNBC cells and TNBC cells treated with angoline in our study, but decreased in MORC2-OV TNBC cells. Moreover, the increased levels of CX43 and cleaved Caspase-3 were inhibited in MORC2-OV TNBC cells treated with Angoline. Besides, Sirt6 has been reported to significantly inhibit the apoptosis of tumor cells [39], and it's expression markedly decreased in shMORC2 TNBC cells and TNBC cells treated with Angoline. However, the decreased level of Sirt6 was hindered in MORC2-OV TNBC cells treated with Angoline. In summary, the alterations in these apoptosis-related proteins suggested that Angoline may induce cell cycle arrest and apoptosis in TNBC cells through its interaction with the MORC2 protein. Therefore, the next flow cytometry assay was performed to verify our speculation. The results showed that the apoptosis rate of TNBC cells dramatically increased after treatment with Angoline; while, the apoptosis rate of MORC2-OV TNBC cells was lower than that of control TNBC cells even both treated with Angoline (Fig. 3E and Fig. S13A).

In addition to inhibiting the MORC2 protein, Angoline has been reported to suppress the IL6/STAT3 signaling pathway [40]. Disruption of the IL6/STAT3 signaling pathway can induce cell cycle arrest and apoptosis, which may contribute to the tumor cell death observed in our study following Angoline treatment. Thus, WB analysis was performed to verify our hypothesis. As shown in Fig. 3F, the level of phosphotyrosine STAT3 (p-STAT3) was significantly reduced in TNBC cells treated with Angoline. Additionally, the cell cycle-related proteins within the IL6/STAT3 pathway exhibited significant changes, including a reduction in c-MYC levels and an upregulation of p21 expression. Furthermore, the expression of PIM1, an anti-apoptotic protein, was significantly reduced in the group treated with Angoline. These changes were also observed in tumor-bearing mice treated with Angoline (Fig. 3G). Thus, Angoline may also killing the TNBC cells by inhibiting the IL6/STAT3 signaling pathway.

In summary, Angoline induces cell cycle arrest in TNBC cells by targeting the MORC2 protein and suppressing the IL6/STAT3 signaling pathway, ultimately triggering cell death through the apoptotic pathway. Thus, Angoline is a promising small molecule inhibitor for targeted therapy in TNBC. Nevertheless, another challenge to address is the development of a more efficient delivery system for Angoline to tumor sites, which could enhance its efficacy and minimize distribution to peripheral organs.

### 2.4 Design and characterization of targeted materials

**Fig. 4:**
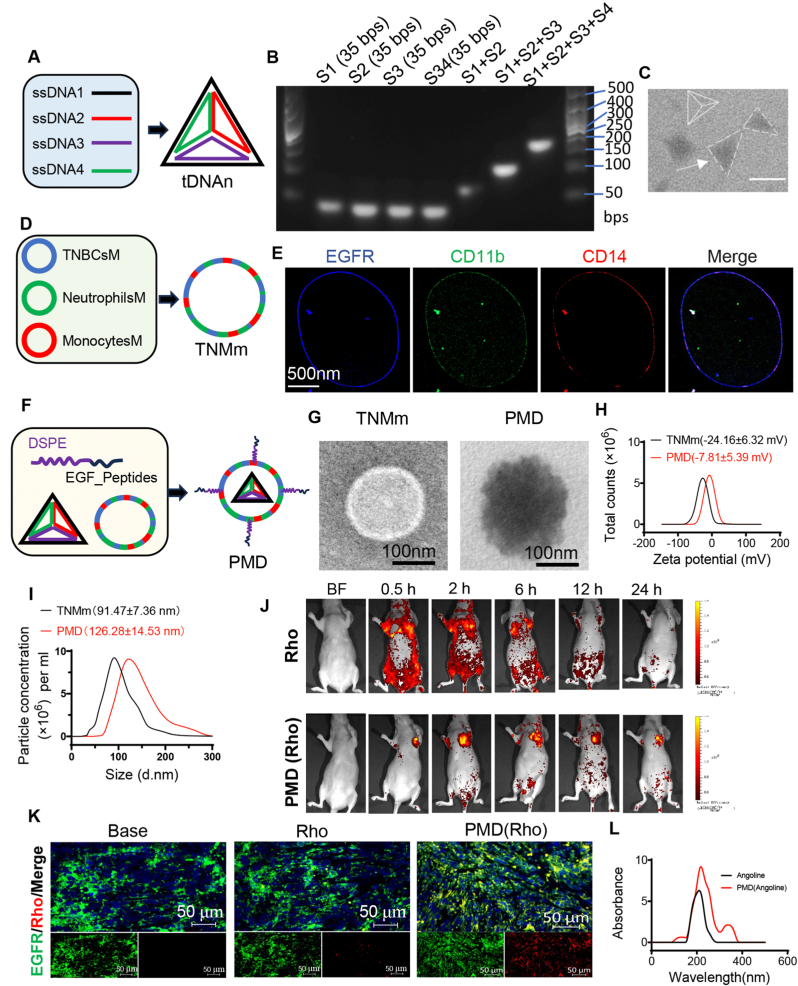
Construction and characterization of biomimetic drug delivery systems (PMD). (A) Preparation of a DNA tetrahedron (tDNAn) formed by annealing four ssDNAs. (B) Agarose gel eletrophoresis of different DNA mixtures, unformed or successfully fabricated tDNAn. Lanes 1, 2, 3 and 4 for ssDNA1, ssDNA2, ssDNA3, ssDNA4, respectively; lane 5: ssDNA1+ssDNA2; lane 6: ssDNA1+ssDNA2+ssDNA3; lane 7: tDNAn. (C) Transmission electron microscopy reveals that tDNAn is tetrahedral in shape (bar, 10 nm). (D) Design of hybrid membrane (TNMm) formed by the cell membranes from TNBC cells, Neutrophils and Monocytes. (E) Immunofluorescence shows that the hybrid membrane is covered with three membrane proteins from TNBC cells, Neutrophils and Monocytes, respectively. (F) Schematic diagram of the preparation process of EGF_Peptides -modified TNMm-enveloped tDNAn. (G) TEM shows the morphologic changes of TMNm after assembled into PMD. (H) The Zeta potential changes of TMNm after assembled into PMD. (I) The particle size changes of TMNm after assembled into PMD. (J) The distribution of PMD(Rho) or Rho in tumor-bearing mice at different time points after ejected via tail vein. (K) The uptake of Rho or PMD(Rho) in TNBC tissue from tumor-bearing mice. (L) UV absorption peak maps of free Angoline or PMD-coated Angoline.

The internal tDNAn (drug-loading core) and heterogeneous TNMm (targeting TNBC, immune evasion, and other functional layers) were assembled into biomimetic drug delivery system (PMD). The internal drug-carrying core mainly consists of four single-stranded complementary base sequences assembled into tDNAn with a tetrahedral structure (Fig. 4A). The successfully step-wise construction of tDNAn with ssDNA1, ssDNA2, ssDNA3, and ssDNA4 was validated by the progressive retardation of different bands in agarose gel electrophoresis (Fig. 4B). Transmission electron microscopy (TEM) revealed that tDNAn was tetrahedral in shape, with size of 10 nm (Fig. 4C). We separately obtained the neutrophil membranes, monocyte membranes, and TNBC cell membranes according to a previously described method [41]. Then, these three membranes were assembled into a hybrid membrane (TNMm) using the membrane extrusion technique at a ratio of 1:1:1 (Fig. 4D). To analyze the total protein composition of TNMm, we examined neutrophil membranes, monocyte membranes, TNBC cell membranes, tDNAn and TNMm by SDS‒PAGE followed by Coomassie blue staining. The results indicated that the protein bands of TNMm matched those of the total protein bands from neutrophil, monocyte, and TNBC cell membranes (Fig. S14A). Previous study have shown that EGFR often exists on the surface of most TNBC cells [42]. Also, CD11b and CD14 are the marker proteins on the surface of neutrophils and monocytes, respectively [43,44]. Therefore, immunofluorescence was employed to detect these specific proteins of TNMm to assess the membrane fusion. As expected, the results showed that the above specific proteins were evenly distributed on the surface of TNMm (Fig. 4E), which indicated that the three cell membranes merged together well. Furthermore, WB was conducted to quantitatively determine the composition of the hybrid membranes. The results indicated that the hybrid membranes consist of 33.14 % TNBC cell membranes, 32.84 % neutrophil membranes, and 34.02 % monocyte membranes (Fig. S14B). To enhance the active targeting capability of TNMm toward TNBC cells, specific EGF functional peptides (EGF_Peptides) binding to EGFR were designed and immobilized into the outer surface of the TNMm via DSPE. Furthermore, to improve the visualization of the biodistribution of the drug delivery system, we labeled the TNMm membrane by conjugating EGF peptides with Rhodamine (Rho; red). The fluorescent scan confirmed that the membrane of PM was labeled with Rho (Fig. S14C).

In order to further assemble the above functional units into biomimetic drug delivery systems, tDNAn, EGF_Peptides and TNMm were extruded to yield biomimetic nanoparticles (PMD) (Fig. 4F). Then, TME was used to observe the changes of membrane morphology after assembling them together. The results showed that the edges of the membrane changed from smooth to jagged (Fig. 4G). Also, the zeta potential has changed from −24.16 ± 6.32 mV to −7.81 ± 5.39 mV because of the positively charged EGF_Peptides (Fig. 4H), which further demonstrated that EGF_Peptides were successfully assembled on the TNMm surface. Moreover, the diameter of the particles also significantly increased, from 91.47 ± 7.36 nm to 126.28 ± 14.53 nm (Fig. 4I). These remarkable changes indicated that EGF_Peptides were successfully assembled around the membrane. Considering that various serum proteins could interfere with PMD function through nonspecific binding, we evaluated the stability of PMD in a culture medium containing in 10 % fetal bovine serum (FBS) PBS at 37 °C. The results demonstrated that PMD maintained its integrity and exhibited excellent stability under these conditions (Fig. S15A). Then, the ability of PMD to resist phagocytosis was further verified by the subsequent experiments. PMD(Rho) and Rho were incubated with neutrophils for a period of time, respectively, and the uptake of PMD(Rho) or Rho was detected by fluorescence microscope. The results showed that neutrophils were entirely covered with red fluorescence in the Rho group, but red fluorescence was rarely seen in the PMD (Rho) group (Fig. S16A). Besides, the differences of uptake between PMD (Rho) and Rho were also seen in monocytes (Fig. S16B). These remarkable results suggested that PMD can efficiently evade the phagocytosis of neutrophils and monocytes. Moreover, PMD(Rho) and Rho were incubated with primary TNBC cells, respectively. The results showed that PMD(Rho) was heavily taken up by TNBC cells, but Rho was rarely taken up by TNBC cells (Fig. S17A). Furthermore, the targeting ability of PMD was evaluated by injecting PMD(Rho) or Rho into the tail vein of TNBC-bearing BALB/c nude mice. As shown in Fig. 4J, Rho rapidly diffused throughout the nude mice and was quickly cleared after injection. By contrast, PMD (Rho) gradually accumulated in the tumor tissue and exhibited slower clearance, indicating that the nanodrug delivery system (PMD) demonstrates effective tumor targeting and prolongs drug retention time. Also, tumor tissue sections confirmed that PMD(Rho) was heavily taken up by tumor cells, but Rho was rarely taken up by tumor cells (Fig. 4K). In summary, the PMD is able to evade the phagocytosis of neutrophils and monocytes and can be easily taken up by TNBC tumor cells, indicating that PMD is an excellent nanomedicine delivery system targeting TNBC tumor tissue. Therefore, PMD(Angoline) was obtained by repeating the PMD preparation process and wrapping Angoline inside the PMD. We compared the UV absorption peaks of single Angoline and PMD (Angoline), and the results showed that Angoline was successfully loaded into tDNAn by insertion between base pairs (Fig. 4L). Furthermore, the encapsulation efficiency (EE) was found to be 72.52 ± 1.36 %, indicating that the PMD system efficiently incorporates Angoline. The drug loading capacity (DLC) of Angoline in the PMD system was determined to be 15.45 ± 0.94 %, demonstrating a reasonable balance between drug incorporation and structural stability of the PMD system. Tumor sites typically present a weakly acidic microenvironment [45]; thus, PBS with varying pH gradients was used to evaluate the release characteristics of PMD (Angoline). The results showed that mild acidity significantly enhanced the release of Angoline from PMD (Angoline), highlighting the potential efficacy of this nanocarrier system in TNBC therapy (Fig. S18A).

A pharmacokinetic (PK) study was performed to assess whether PMD could improve Angoline's systemic circulation and extend its half-life. Following a single intravenous injection of various formulations into Sprague-Dawley (SD) rats, blood samples were collected at predetermined time points and analyzed using HPLC. As illustrated in Fig. S19A and summarized in Table 1, the plasma concentration-time curves and PK parameters are presented. Compared to the rapid clearance of Angoline (t₁/₂: 4.15 ± 0.06 h), PMD (Angoline) demonstrated a significantly prolonged circulation time in the blood (t₁/₂: 29.85 ± 0.35 h). Furthermore, the data obtained from the area under the concentration-time curve (AUC) further confirmed the enhanced PK properties. Thus, the biomimetic nanodrug delivery system (PMD) confers Angoline with enhanced PK properties, attributed to its high stability and sustained drug release kinetics.

**Table 1:** Pharmacokinetic parameters of Angoline and PMD (Angoline).

PK parameter	Drug formuations	
	Angoline	PMD (Angoline)
t_1/2_ (h)	4.15 ± 0.06	29.85 ± 0.35
AUC_(₀₋ₜ)_ (ug.h/ml)	13.16 ± 0.32	725.37 ± 0.57
AUC_(₀₋inf)_ (ug.h/ml)	15.43 ± 0.19	826.25 ± 0.71
CL (ml/h)	137.27 ± 26.35	1.31 ± 0.03
Vd (ml/h)	835.41 ± 119.28	56.37 ± 1.86
MRT (h)	6.08 ± 0.18	43.03 ± 0.74
C_max_ (ug/ml)	3.41 ± 0.15	105 ± 1.48
C_0_ (ug/ml)	3.67 ± 0.28	110.53 ± 1.87

t1/2, half-life time; AUC, the area under the plasma concentration-time curve; AUC(0-t), AUC from 0 to time t; AUC(0-inf), AUC extrapolated to infinity; CL, total clearance of the drug from plasma after intravenous administration; Vd, volume of distribution; MRT, mean residence time; C, drug concentration. Data are presented as the means ± SD (n = 5).

### 2.5 Angoline cytotoxicity was enhanced by PMD in TNBC tumors

**Fig. 5:**
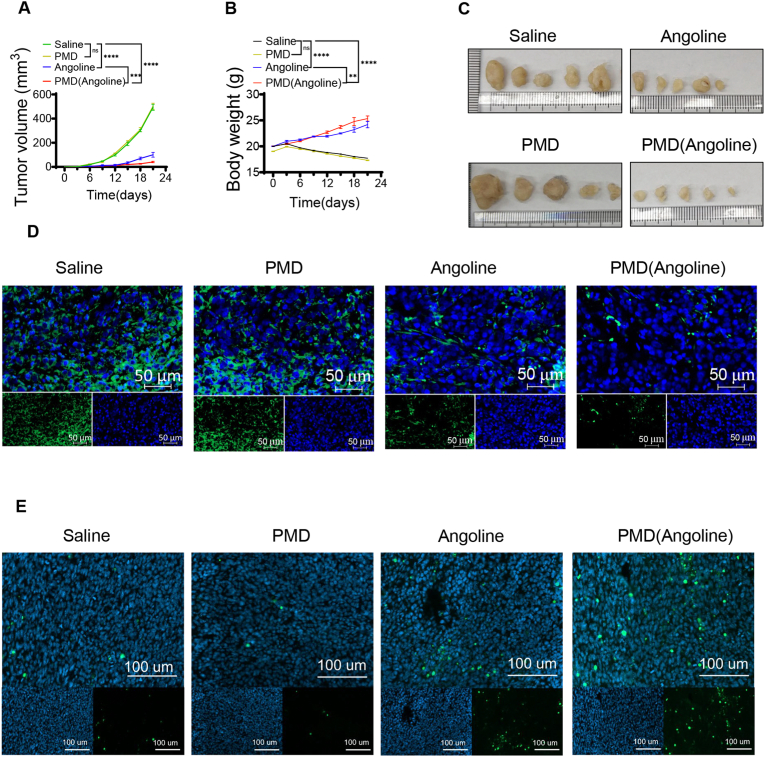
The biomimetic drug delivery systems (PMD) can enhance the inhibiting effect of Angoline on growth of TNBC in vivo. (A) Tumor volume growth curves of mice treated with different drugs. (B) Body weight curves of mice treated with different drugs. (C) Images of xenograft tumors from mice treated with different drugs. (D) Immunofluorescence results of Ki-67 expression in tumor tissue of mice in different treatment groups. (E) TUNEL staining to evaluate apoptosis of TNBC cells in mice treated with different drugs. ∗P < 0.05; ∗∗P < 0.01; ∗∗∗P < 0.001; ∗∗∗∗P < 0.0001; ns, no significance.

Given the highly effective killing effect of Angoline on TNBC cells and the excellent capacity of PMD targeting TNBC, we further examined whether PMD could enhance the inhibitory effect of Angoline on TNBC tumors. Xenograft tumor models were established by injecting primary TNBC cells under the skin of BALB/c nude mice. Then, one week after implantation, the mice were administered with normal saline, Angoline, PMD and PMD (Angoline) respectively via tail vein injection every 3 days. Also, the tumor volume in mice was measured every three days. The group receiving PMD treatment presented a similarly rapid tumor growth compared with the saline-treated group. Encouragingly, the administration of both Angoline and PMD(Angoline) contributed to significant suppression of the primary tumor growth. Notably, PMD(Angoline) exhibited stronger effect, inhibiting progression of the tumors compared with Angoline (Fig. 5A). In addition, the body weight of each mouse was continuously monitored, and the average body weight was gradually decreased in the saline and PMD groups (Fig. 5B). By contrast, the body weight significantly increased in other two groups, especially in mice treated with PMD(Angoline). This dramatic trend of weight change revealed that effective antitumor therapy of PMD(Angoline) can improve the physical condition of tumor-bearing mice. Finally, all mice were sacrificed three weeks later and xenografts were removed. Consistent with tumor growth curve, the tumor volume in the Angoline group was significantly smaller than that in the saline or PMD groups but bigger than that of PMD(Angoline) group (Fig. 5C). Additionally, significant differences in tumor weight were observed among the four groups (Fig. S20A). Ki67 is a well-recognized biomarker for cancer cell proliferation, and a reduction in Ki67 % indicates that the candidate drug has better anti-tumor properties [46]. Thus, we performed immunofluorescence staining to analyze the expression of Ki-67 in different groups. The results showed that the proliferation capacity of tumor cell was significantly lower in the Angoline group than that in saline or PMD groups, but higher than PMD(Angoline) group (Fig. 5D). Furthermore, TUNLE staining was performed to evaluate the apoptosis of tumors treated with different drugs (Fig. 5E). The group treated with Angoline exhibited a significantly higher apoptosis rate compared to the groups treated with saline or PMD. Moreover, PMD (Angoline) exhibited a stronger pro-apoptotic effect than Angoline, indicating that PMD enhanced the tumor killing effect of Angoline. Taken together, these findings suggested that Angoline coated with PMD can enhance its anti-cancer effect in TNBC.

**Fig. 6:**
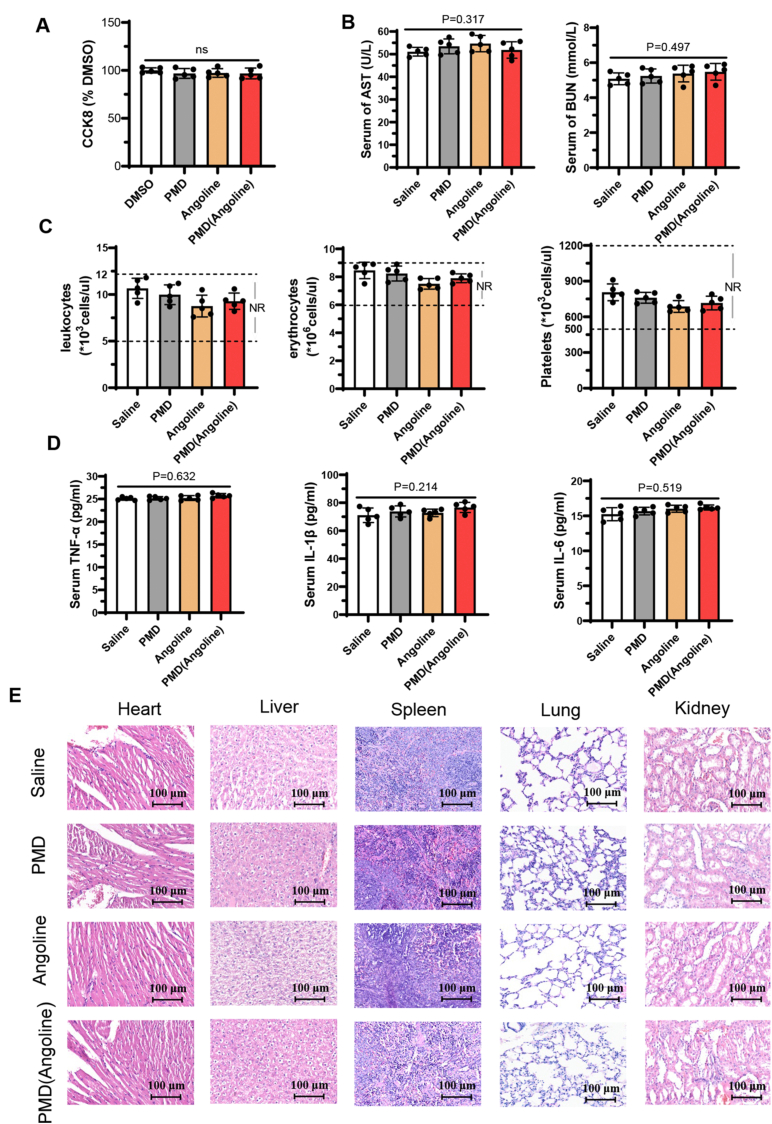
Toxicity tests of PMD and PMD (Angoline) at cellular and animal levels. (A) The toxicity of the drugs on the normal breast epithelial cells (MCF-10A). (B) Serum AST and BUN levels in mice treated with different drugs. n = 5 biologically independent samples. (C) The levels of leukocytes, erythrocytes, and platelets in mice treated with different drugs. n = 5 biologically independent samples. (D) The levels of serum TNF-*α*, IL-1*β*, and IL-6 in mice treated with different drugs. n = 5 biologically independent samples. (E) HE staining results of vital organs (heart, liver, spleen, lung and kidney) from mice treated with different drugs. ns, no significance.

Furthermore, the toxicity and side effects of PMD (Angoline) were evaluated through a comprehensive series of experiments. Firstly, normal breast epithelial cells (MCF-10A) were used to assess drug toxicity. As shown in Fig. 6A, neither Angoline nor PMD (Angoline) exhibited toxicity toward MCF-10A cells, demonstrating the safety of PMD (Angoline) for normal breast tissue. Then, as shown in Fig. 6B, there were no significant differences in AST and BUN levels among the four groups, indicating that PMD (Angoline) does not have a notable impact on liver or kidney function. Then, leukocytes, platelets, and erythrocytes were in the normal ranges among all the groups (Fig. 6C), suggesting that PMD (Angoline) has no obvious hematotoxicity. Furthermore, we tested the immune-promoting cytokines, such as TNF-*α*, IL-1β and IL-6. The results showed no significant changes among the four groups (Fig. 6D), indicating that PMD (Angoline) exhibits very low immunogenicity. Finally, HE staining showed that PMD (Angoline) did not affect the health status of key organs in mice (Fig. 6E).

## 3 Conclusions

MOCR2 is highly expressed in TNBC compared with normal breast tissue, which also contributes to worse outcome for patients with TNBC. Additionally, MOCR2 promotes the progression of TNBC by accelerating the cell cycle, indicating that MOCR2 may be a potential therapeutic target for TNBC. Therefore, high-throughput screening was performed to find the effective small molecule compounds that interact with MORC2 protein and inhibit the growth of TNBC cells. Encouragingly, the results demonstrated that Angoline binds strongly to the MORC2 protein and inhibits its function, resulting in inhibiting the growth of TNBC by blocking the cell cycle and inducing the apoptotic pathway. Furthermore, our designed precision targeted therapy system (PMD) can efficiently evade the phagocytosis of immune system and target TNBC tissue, which will significantly enhance the killing effect of Angoline on TNBC tumors.

## 4 Materials and methods

### 4.1 Human samples and cell lines

After approved by the Human Tissue Research Ethics Committee of Affiliated Hospital of North Sichuan Medical College and receiving the informed consent from all patients, we obtained tumor tissue and adjacent nontumor tissue from breast cancer patients and abided by the ethical guidelines for research on all human subjects. Neutrophils and monocytes were isolated from human (TNBC patients) donor-derived peripheral blood according to the methods reported by previous researches [47,48]. These obtained specimens were kept under suitable conditions and used in subsequent experiments. Moreover, human TNBC cell lines (MDA-MB-231, MDA-MB-468 and BT-549) and normal breast epithelial cell line (MCF-10A) were purchased from Procell (Wuhan, China). All cell lines were tested for mycoplasma, chlamydia and fungi before the experiment, and were confirmed negative to ensure the accuracy and reliability of the experiment.

### 4.2 High-throughput virtual screening of small molecule compounds

Topscience Database, containing more than 2 × 10^5^ small molecule compounds, was used to screen out the potential small molecule compounds targeting MORC2 protein. The small molecule compounds were hydrogenated, desalted and adjusted charge. Also, the molecular force field was applied to obtain the required 3D conformation of small molecule compounds by energy minimization. The structure of the MORC2 protein (ID: 5OF9) was downloaded in PDB format from the PDB Protein database (https://www.rcsb.org/). Then, the downloaded structure was imported into Discovery Studio 2019 Client and processed by visualization and correction. Moreover, the pre-treated MORC2 protein and small molecule compounds of Topscience Database were imported into the docking module for quick docking. Finally, the top 500 small molecule compounds with docking scores were selected from the Topscience Database according to the screening results of docking scores. In addition, the display of proteins and small molecules was adjusted as needed to obtain a clear binding image, and the visualizations were saved for subsequent analysis and display.

### 4.3 Synthesis and characterization of tDNAn

In our study, the design and construction of tDNAn were performed according to previous reports [49]. First, four ssDNAs (S1–S4) were added into TM buffer (10 mM Tris-HCl, 5 mM MgCl2, pH 8.0) and mixed evenly at a final concentration of 1 μM. Then, the mixture was heated at 95 °C for 10 min and immediately cooled to 4 °C for 20 min via a PCR thermal cycler (Jena Bioscience, German). Ultimately, the synthesized products were tDNAn. Moreover, the relative molecular mass distribution was detected using agarose gel electrophoresis to confirm the successful construction of tDNAn with four ssDNAs. The size and zeta potential of tDNAn were analyzed by DLS on Zeta-sizer Nano ZS90 (Malvern Instrument, Ltd.). TEM was used to observe the morphological characteristics and particle stability of the tDNAn. A drop of the tDNAn was dripped onto cleaved mica, and then TEM images were captured after the sample dried.

### 4.4 Preparation of hybrid membrane (TNMm)

The cell membrane materials were derived from TNBC cells, neutrophils and monocytes, which were extracted from human samples as mentioned above, and they were hybridized together according to previously reported study [41]. Briefly, the collected cells were suspended in a buffer for extracting membrane proteins and incubated in an ice bath for 15 min. After broken via freeze–thaw method, the mixture was centrifuged at 3000 rpm for 10 min at 4 °C, and the collected suspension was centrifuged at 12 000 rpm for 40 min at 4 °C to obtain membrane fragments. Subsequently, the three obtained membrane fragments were mixed in a ratio of 1:1:1 and sonicated in an ice-water bath for 10 min. The fused hybrid cell membranes were then extruded through 800, 400, 200, and 100 nm polycarbonate porous membranes to achieve the uniform size of TNMm, as reported in a previous study [50]. Confocal microscopy was used to examine the distribution of the three cell membranes with unextruded TNMm based on their specific proteins, including EGFR, CD11b, and CD14. Meanwhile, SDS-PAGE was conducted to analyze the differences in protein composition between TNMm and the other three cell membranes. Moreover, in order to enhance the tumor targeting effect of TNMm, EGF_Peptides were designed and attached to the cell membranes by amidation with DSPE. Also, Rho was modified on EGF_Peptides via amidation to better characterize the membrane system, and the labeling effect was confirmed by fluorescence microscopy.

### 4.5 Statistical analysis

In our study, all experiments were carried out in triplicates, and the quantitative data are presented as the means ± standard deviations (SD). Student's unpaired *t*-test was used to compare the difference between two groups, and one-way analysis of variance (ANOVA) was conducted to estimate the difference among multiple groups. All statistical analysis were performed with the GraphPad Prism 8 software, and a p-value <0.05 was considered statistically significant.

More detailed methods are provided in the Supporting Information.

## CRediT authorship contribution statement

**Xiaohan Su:** Writing – original draft, Investigation. **Yunbo Luo:** Writing – original draft, Investigation, Data curation. **Yali Wang:** Software, Methodology, Data curation. **Peng Qu:** Formal analysis, Data curation. **Jun Liu:** Software. **Shiqi Han:** Methodology. **Cui Ma:** Formal analysis, Data curation. **Shishan Deng:** Writing – review & editing, Supervision. **Qi Liang:** Writing – review & editing, Supervision. **Xiaowei Qi:** Writing – review & editing, Funding acquisition. **Panke Cheng:** Writing – review & editing, Funding acquisition, Conceptualization. **Lingmi Hou:** Writing – review & editing, Funding acquisition, Conceptualization.

## Declaration of competing interest

The authors declare that they have no known competing financial interests or personal relationships that could have appeared to influence the work reported in this paper.

## Data Availability

Data will be made available on request.
