# Supplementary material for: A select inhibitor of MORC2 encapsulated by chimeric membranecoated DNA nanocage target alleviation TNBC progression
Source: Mater Today Bio. 2025 Jan 19;31:101497. doi: 10.1016/j.mtbio.2025.101497 (PMC11791359; doi:10.1016/j.mtbio.2025.101497)
Supplement: Multimedia component 1 [file mmc1.docx]

**Supplementary information**

**A select inhibitor of MORC2 encapsulated by chimeric membranecoated DNA nanocage target alleviation TNBC progression**

Xiaohan Su^1,2^*, Yunbo Luo^1,3^*, Yali Wang^3^*, Peng Qu^4^, Jun Liu^5^, Shiqi Han^3^, Cui Ma^6^, ShishanDeng^3^, Qi Liang^4^, Xiaowei Qi^7#^, Panke Cheng^5,8#^, Lingmi Hou^1#^

1. Breast Surgery, Sichuan Cancer Hospital & Institute, Sichuan Cancer Center, School of Medicine, University of Electronic Science and Technology of China, Chengdu, China

2. Breast Surgery Department, The 404th Hospital of Mianyang, Mianyang, China

3. Department of Breast and thyroid Surgery, Biological targeting Laboratory of Breast cancer, Academician (expert) workstation, Affiliated Hospital of North Sichuan Medical College, Nanchong, China

4. Department of Laboratory Medicine, Affiliated Hospital of North Sichuan Medical College, Nanchong, China

5. Institute of Cardiovascular Diseases & Department of Cardiology, Sichuan Provincial People's Hospital, School of Medicine, University of Electronic Science and Technology of China, Chengdu, China

6. Department of Mathematics, Army Medical University, Chongqing, China

7. Department of Breast Surgery, Southwest Hospital, Army Medical University, Chongqing, China

8. Ultrasound in Cardiac Electrophysiology and Biomechanics Key Laboratory of Sichuan Province, Chengdu, China

^*^These authors contributed equally.

# ***Correspondence to:*** Lingmi Hou (Email: houlingmi@163.com), Panke Cheng (E-mail: [chengpk1002@163.com](mailto:chengpk1002@163.com)) and Xiaowei Qi (E-mail: qxw9908@foxmail.com).

**Supplementary Materials and Methods**

***Cell culture***

MDA-MB-231 cells were cultured in DMEM containing 10% fetal bovine serum (FBS; AC03L055, LIFE iLAB BIO, Shanghai, China) and 1% penicillin/streptomycin (15140-122, gibco, China) at 5% CO2 and 37 °C. MCF-10A, MDA-MB-468 and BT-549 cells were cultured with their special culture medium purchased from Procell (Wuhan, China). The MORC2 knockdown /overexpression lentivirus and negative control virus were purchased from GeneChem (Shanghai, China). Then these lentivirus and negative control virus (MOI = 10) were transfected into MDA-MB-231 and BT-549 cells using polybrene (Beyotime Biotechnology, China) according to the manufacturer’s protocol.

***CCK-8 assay***

MDA-MB-231 and MDA-MB-468 cells were inoculated on 96-well plates with 5×10^3^ cells per well, and then different small molecule drugs were added according to study requirements after the cell culture reached the appropriate density. Twenty-four hours after treatment, CCK-8 reagent was added to each well and incubated for 2 hours. Then, the absorbance of each well was measured at 450 nm using an enzyme-labeler to calculate the relative survival rate of cells.

***Trypan blue staining and YO-PRO-1 staining experiment***

MDA-MB-231 and MDA-MB-468 cells were inoculated on 6-well plates with 2×10^5^ cells per well, and different drugs were added to the plates after the cells reaching appropriate density. The cells were stained with trypan blue staining cell viability assay kit (Beyotime Biotechnology, China) or Green fluorescent probe of apoptotic and necrotic cells (YO-PRO-1, Beyotime Biotechnology, China) 24 hours after treatment. Images of the cells were then observed under an ordinary light microscope. The cell images stained with trypan blue were analyzed with ImageJ software, the number of living and dead cells was counted, and the cell survival rate was calculated. In YO-PRO-1 staining experiment, cell images were observed and taken under fluorescence microscopy.

***Transwell migration assay***

To synchronize the cells in G0/G1 phase, TNBC cells were cultured with serum-free medium for 36 hours. Meanwhile, the upper chamber of a 24-well plate was coated with Matrix gel (Corning, 356231) to mimic the extracellular matrix (ECM) of the tumor microenvironment. Then, 200 µL serum-free medium containing 2 × 10^5^ cells were added into the upper chamber. The bottom chamber contained growth medium with 10% FBS. After incubated at 37 °C for 24 hours, the migrated cells at bottom of the upper chamber insert was fixed with 100% methanol and stained with crystal violet. The cells on the inner membrane of the upper chamber were gently removed. The images of invaded cells were captured and analyzed.

***Western blotting assay***

TNBC cells were inoculated in 10-cm plates with 50–60% confluence and incubated for 24 hours. Then, after cleaning the cells with PBS three times, the total proteins of cells were harvested by adding the RIPA lysis buffer and 1% Phenylmethanesulfonyl Fluoride (PMSF) into the plates and scraping off the lysed cells. The total proteins were calculated by the BCA protein estimation assay and were isolated by 10% SDS‒PAGE. The protein was transferred onto the PVDF membrane and then blocked with 5% skim milk powder for 2 hours. The protein bands were probed with corresponding antibodies and incubated at 4 °C overnight. Then, the secondary antibodies were added and incubated 1 h at room temperature. Finally, the membranes were detected by the enhanced chemiluminescence detection system.

***Immunofluorescence microscopy (IF) of formalin fixed, paraffin embedded (FFPE) tissues***

After fixed in 4% paraformaldehyde for 48 hours, the tissues were embedded with paraffin. Then, tissue sections (4 μm) were prepared using a paraffin microtome to assess the expression of target proteins. Following deparaffinization and rehydration, the antigens were retrieved with citrate buffer (pH = 6.0). Tissue sections were penetrated with 0.5% Triton X-100 and incubated with pure goat serum at room temperature for 30 minutes. The primary antibody was incubated with the tissue overnight at 4°C, followed by incubation with the corresponding fluorescent secondary antibody at 37°C for 1 hour. After incubation of DAPI dye at 37 °C for 20 minutes, the tissue sections were sealed with antifade mounting medium. Ultimately, images were obtained using the fluorescence microscope and analyzed by Image J software.

***Immunohistochemistry (IHC) of tissues***

Tissue sections were obtained according to the above experimental methods and the antigens were retrieved with citrate buffer (pH = 6.0). After treatment with peroxidase blocking solution (3% H₂O₂) for 10 minutes at room temperature, the tissues were incubated with anti-MORC2 antibody at the recommended dilution overnight at room temperature. Then, an HRP-conjugated secondary antibody was added and incubated 30 minutes. Then, the sections were stained with DAB for about 4 minutes at room temperature, counterstained with hematoxylin and subjected to alcohol dehydration. Finally, the IHC results were estimated using a microscope.

***Measurement of cell cycle and apoptosis by flow cytometry***

The treated cells were digested with pancreatic enzymes and then were collected and washed with PBS. The cells were immobilized with pre-cooled 70% ethanol at 4 ℃ for 12-24 hours, and then centrifuged, washed, and suspended to ensure uniform cell dispersion. Propyridine iodide staining solution was added to each cell sample and incubated without light at 37 ℃ for 30 minutes. Subsequently, the labeled cells were subjected to cell cycle analysis using flow cytometer (Beckman Coulter, USA), and the results were processed by FlowJo software. For the apoptosis assay, 1 × 10^5^ cells per sample were collected into 1.5 mL centrifuge tubes and were re-suspended with 195 μL Annexin V-FITC binding solution. Then, 5 μL Annexin V-FITC and 10 μL PI dyeing solution were added into every sample to re-suspend the cells and incubated at 20-25ºC for 20 minutes. Flow cytometry was used to detect red fluorescence at 638 nm and green fluorescence at 488 nm, and the apoptosis results were analysed by FlowJo software.

***Animal assay***

All animal studies complied with relevant regulations, and all experimental procedures involving mice were approved by the Animal Care Committee of North Sichuan Medical College. Female BALB/c nude mice aged 6-8 weeks (weight 18-20 g) were purchased from Chengdu Dashuo Laboratory Animal Co., LTD. Before the animal experiments, TNBC cells (stably transfected with the sh-NC/sh-MOCR2 lentivirus) were suspended with PBS and adjusted to the concentration of 1×10^6^ cells/100 μL. Then, 100 μL of cell suspension was subcutaneously grafted in the left flank of each mouse. After the xenografts forming, the volume of xenografts and the weight of mice were continuously monitored. After 3 weeks, the mice were sacrificed, and xenografts were removed to assess tumor growth across the different groups.

***Purification of MORC2 protein***

The sequence of human MORC2 protein gene was retrieved from Pubmed and cloned into pET28a vector containing 6 His tags. Subsequently, it was transformed in Escherichia coli BL21 and induced to express by IPTG under suitable condition. Bacterial precipitates were collected and MORC2 protein was adsorbed by Ni-NTA purification column. Binding buffer buffers were used to wash the miscellaneous proteins and protein elution was performed using gradient elution (20 mM to 500 mM imidazole) to obtain the purified MORC2 protein. SDS-PAGE gel and Coomassie bright blue staining were performed to explore the optimal conditions for MORC2 protein expression and purification, including IPTG concentration, temperature and duration of protein induction, Binding buffer washing times and Imidazole elution concentration.

***Surface plasmon resonance (SPR) molecular interaction experiment***

The BCA kit was used to detecting the concentration of purified MORC2 protein to ensure that the protein amount is adequate and accurate. Next, MORC2 protein was biotinylated according to the instructions of G-MM-IGT Biotinylation Kit and coupled to biotinylated sensors. Small molecule compounds with different concentration gradients (100 nM, 50 nM, 25 nM, 12.5 nM, 6.25 nM, 3.125 nM) were injected. The affinity constant between the small molecule compound and MORC2 protein was obtained from the signal value at the time point 2 seconds before the end of injection.

***Synthesis and characterization of the*** ***biomimetic drug delivery systems (PMD)***

The TNMm-cloaked tDNAn (PMD) were finally acquired by mixing tDNAn with TNMm, sonicating them for 10 min in an ice-water bath, and then extruding them through 800, 400, and 200 nm polycarbonate porous membranes. Then, the mixture suspension was centrifuged at 12 000 rpm 4 °C for 25 min to remove unassembled cell membranes and tDNAn, and the obtained PMD were resuspended in 10 mM HEPES buffer at pH 7.4 for next experiments. Finally, TEM and Zetasizer Nano ZS 90 were used to observe the morphological structure and size of the PMD.

***Capacity of immune escape and targeting tumor tissue for PMD***

In order to estimate the capacity of PMD escaping the phagocytosis by blood immune cells (neutrophil and monocyte), PMD(Rho) or Rho were incubated with immune cells for 4 hours, respectively. Then, fluorescence microscopy was used to detect the phagocytosis of PMD(Rho) or Rho by immune cells. Moreover, PMD(Rho) or Rho were incubated with primary TNBC cells for 6 hours, and then the uptake of PMD(Rho) or Rho by TNBC cells was examined via fluorescence microscopy, which can reveal the ability of PMD targeting tumor tissue. Additionally, PMD(Rho) or Rho were injected into tumor-bearing mice via tail veins, and then fluorescent in vivo imaging was performed to determine the distribution of Rho or PMD(Rho). Finally, the mice were sacrificed and tumors were removed to observe the uptake of PMD(Rho) or Rho by TNBC cells through detecting the fluorescence intensity of Rho and EGFR.

***In vivo antitumor efficacy evaluation of PMD-encapsulated Angoline***

PMD(Angoline) was prepared by encapsulating Angoline into tDNAn following the assembly steps mentioned above. Tumor-bearing mice were established using primary TNBC cells obtained from patients, following the methods described in the animal assessment section. Then, those mice were intravenously administrated with saline, Angoline, PMD and PMD(Angoline) every 3 days, respectively. Meanwhile, the tumor volume and mice weight were continuously monitored every 3 days. Ultimately, the mice were sacrificed after three weeks to obtain the tumors and vital organs (heart, liver, spleen, lung and kidney). Ki-67 expression was detected by immunofluorescence to determine the tumor growth in different groups. Also, HE staining was used to determine the damage of different drugs to important organs.

**Supplementary Figures**


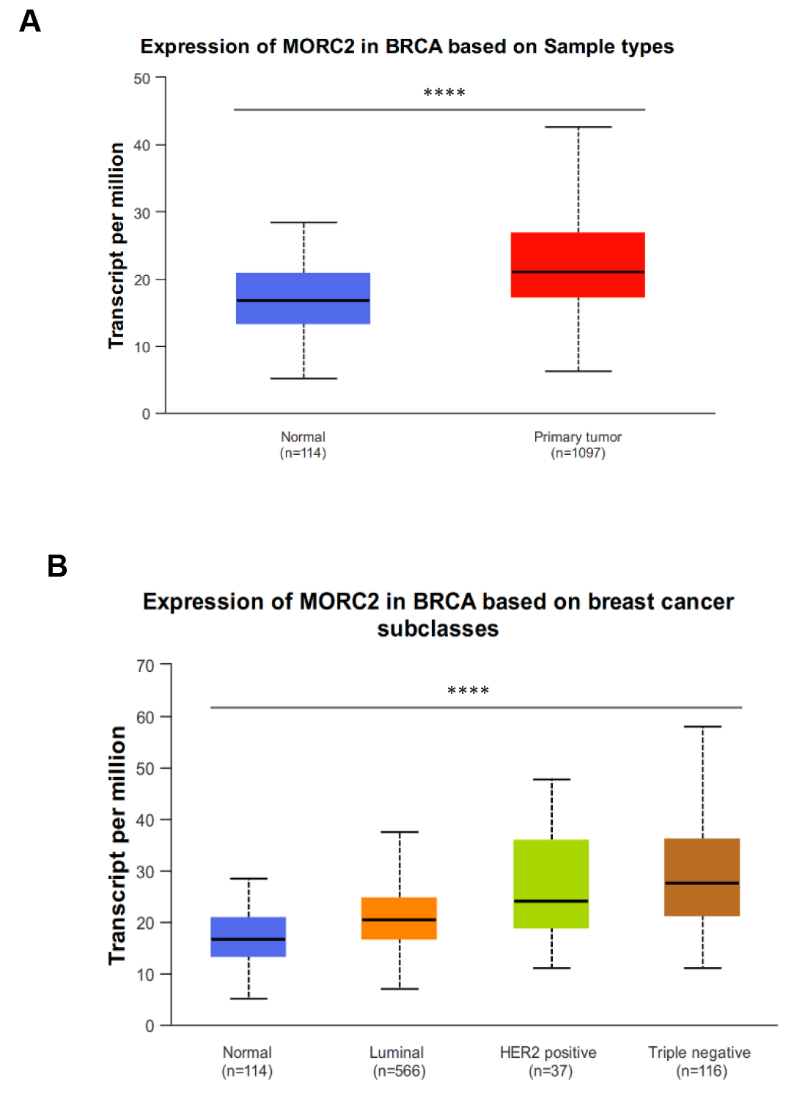


Fig S1. MORC2 is markedly upregulated in breast cancer, especially in triple-negative breast cancer (TNBC). (A) The expression difference of MORC2 between normal breast tissue and primary breast cancer tissue from the UALCAN Database. (B) The expression difference of MORC2 between normal breast tissue and different molecular types of breast cancer tissue from the UALCAN Database. **** P < 0.0001.


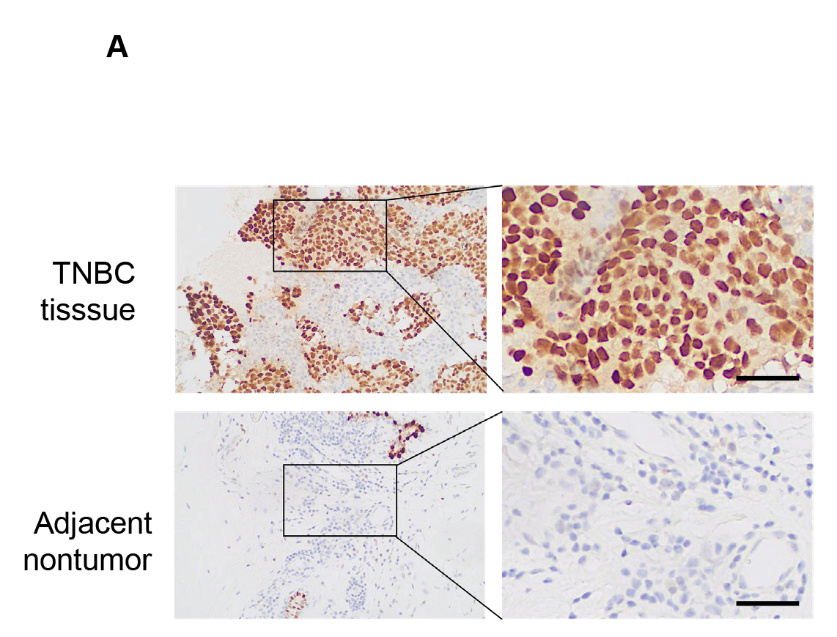


Fig S2. (A) IHC staining of MORC2 expression in TNBC paraffin-embedded specimens and adjacent nontumor tissue (bar, 50um).


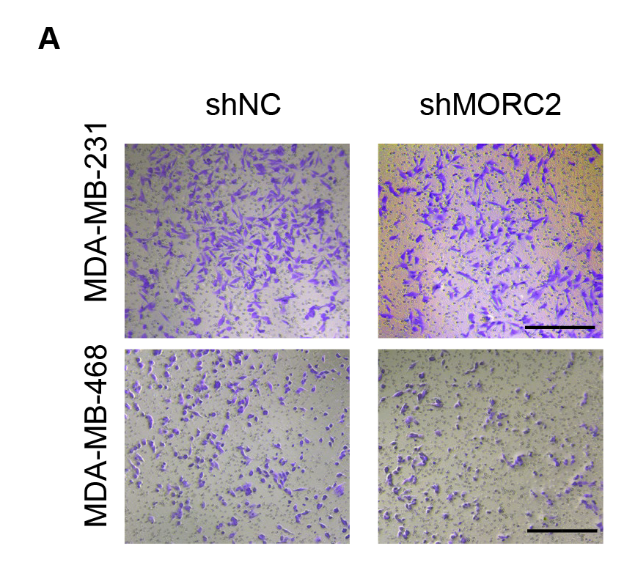


Fig S3. (A) Micrographs of the invasiveness of TNBC cells in the matrigel-coated transwell assay (bar, 50μm).


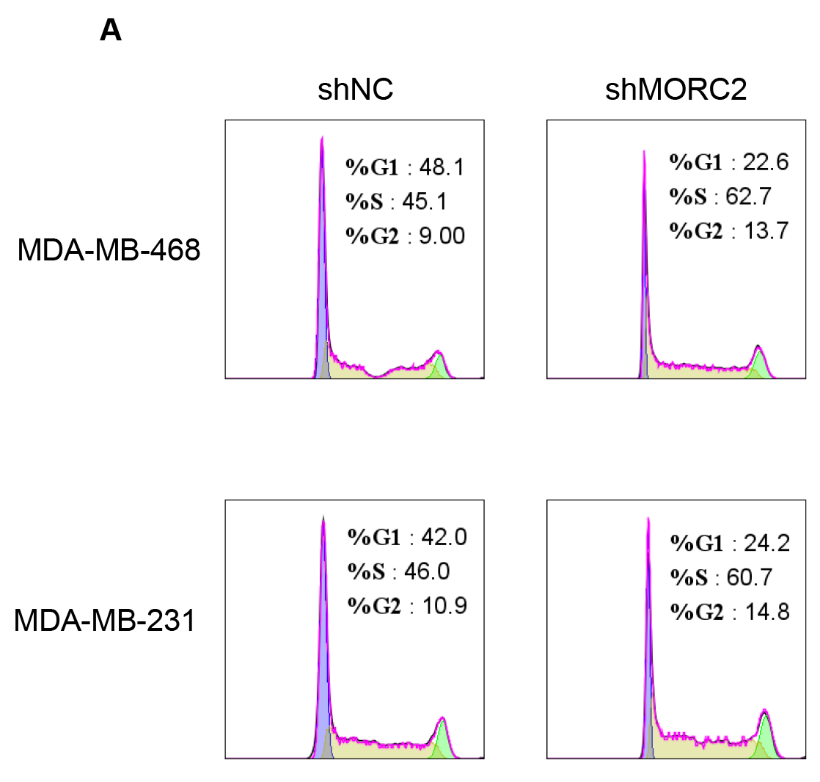


Fig S4. (A) The cell cycle distribution of TNBC cells before and after MORC2 knockdown.


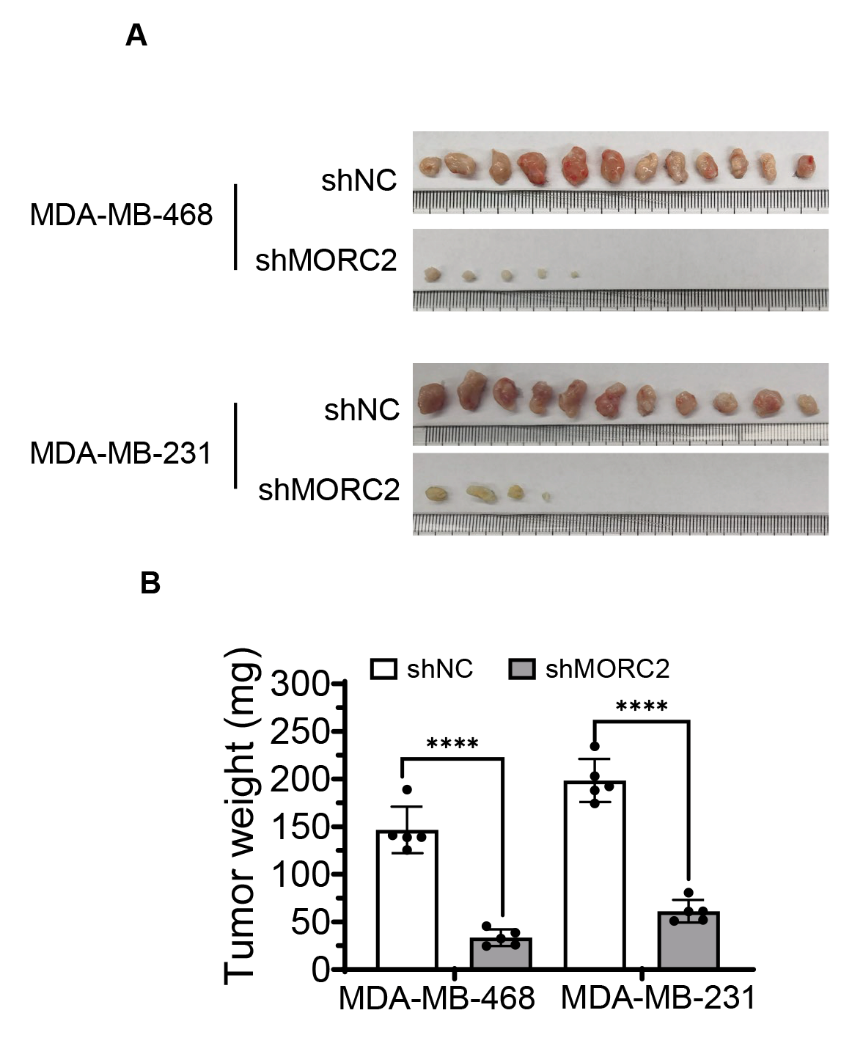


Fig S5. (A) Images of xenograft tumors from shNC and shMORC2 groups harvested at the endpoint. (B) The weight of inoculated xenograft tumors in shMORC2 or shNC groups.


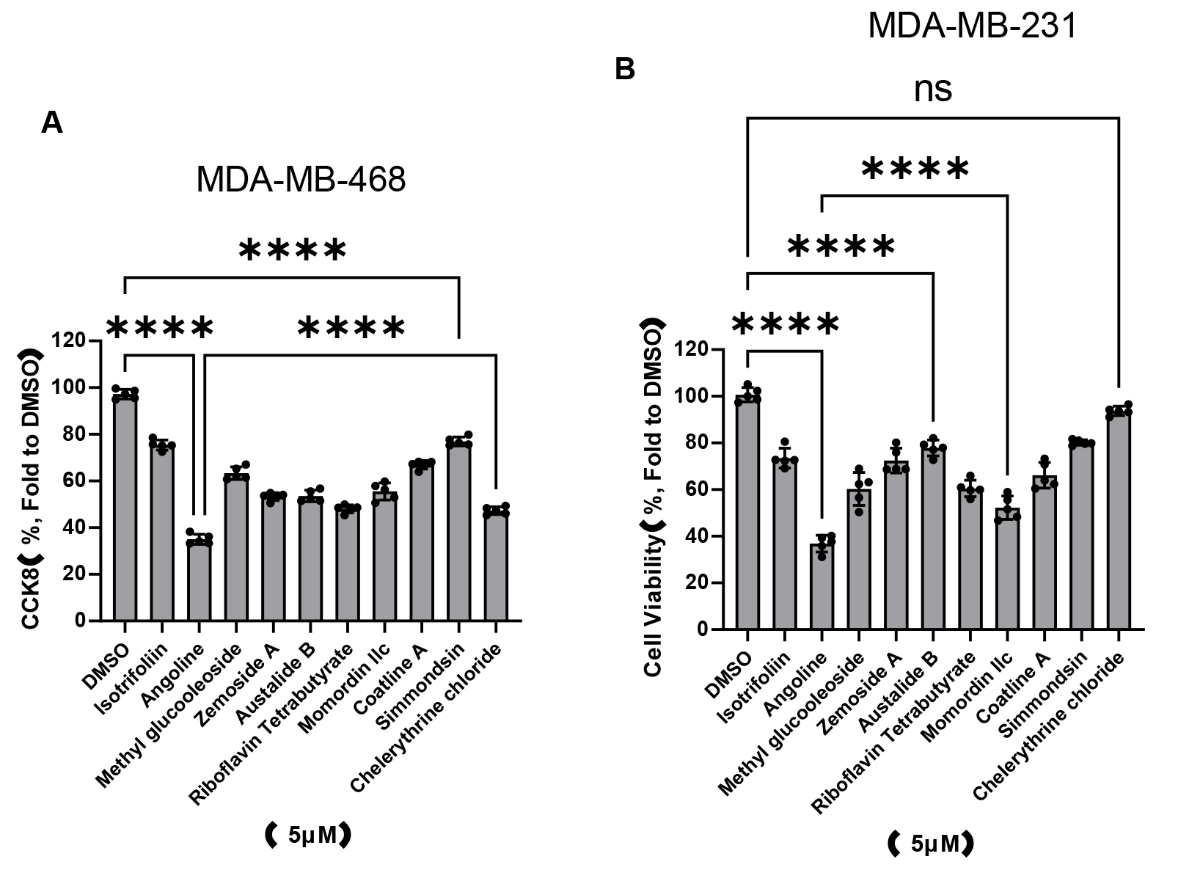


Fig S6. CCK8 assay was performed to verify the killing effect of the top 10 small molecule compounds on MDA-MB-468 cells (A) and MDA-MB-231 cells (B). **** P < 0.0001; ns, no significance.


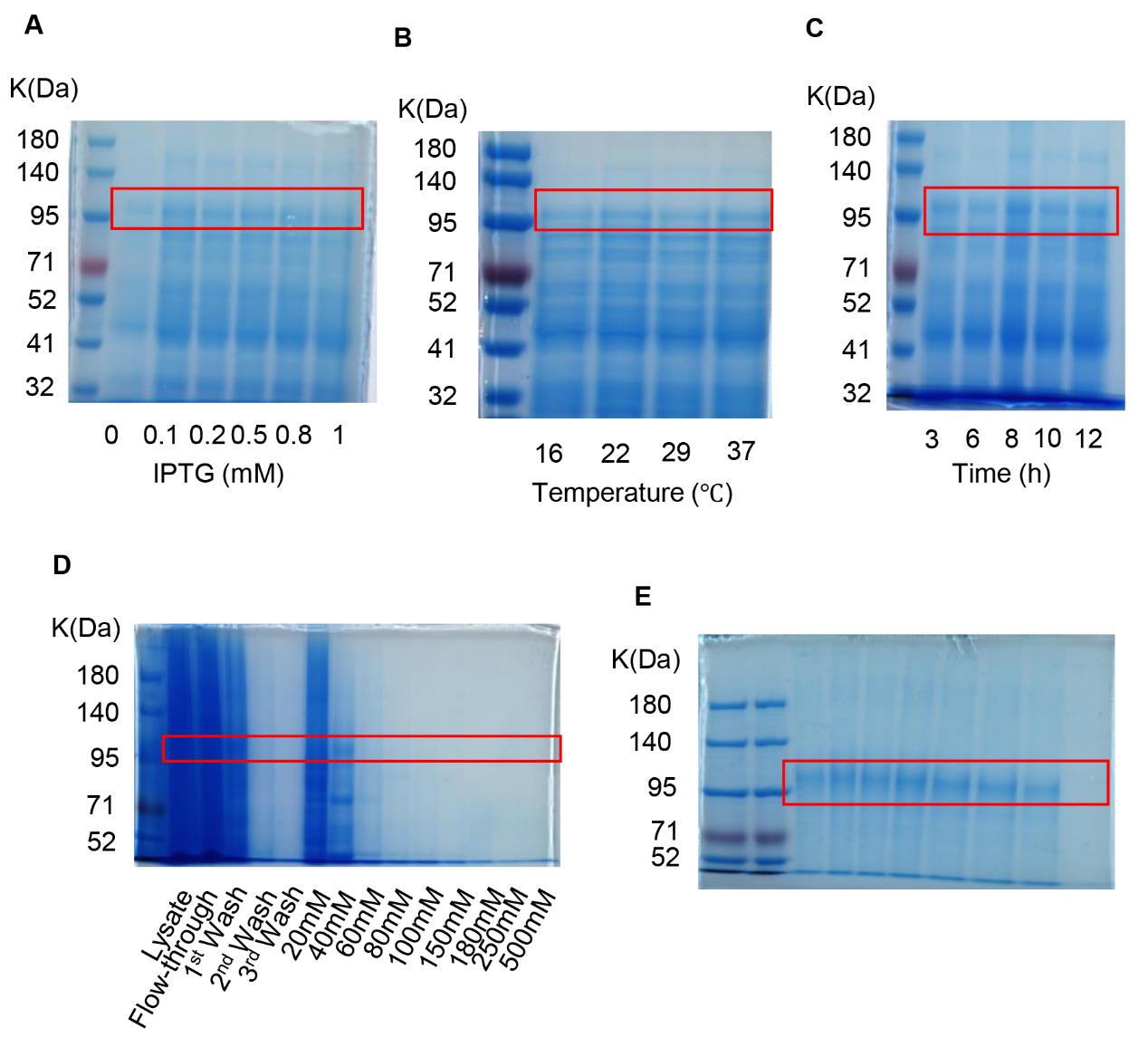


Fig S7. Exploration of the optimal conditions for purification of MORC2 protein by SDS-PAGE gel and Coomassie bright blue staining. (A) MORC2 protein expression of recombinant plasmid induced by different concentrations of IPTG. (B) MORC2 protein expression of recombinant plasmids induced by IPTG at different temperatures. (C) MORC2 protein expression of recombinant plasmid induced by IPTG in different duration. (D) Schematic diagram of Binding/Wash Buffer washing steps and optimal imidazole elution concentration optimization in MORC2 protein purification. (E)The purified MORC2 protein was obtained and verified by Coomassie bright blue staining.


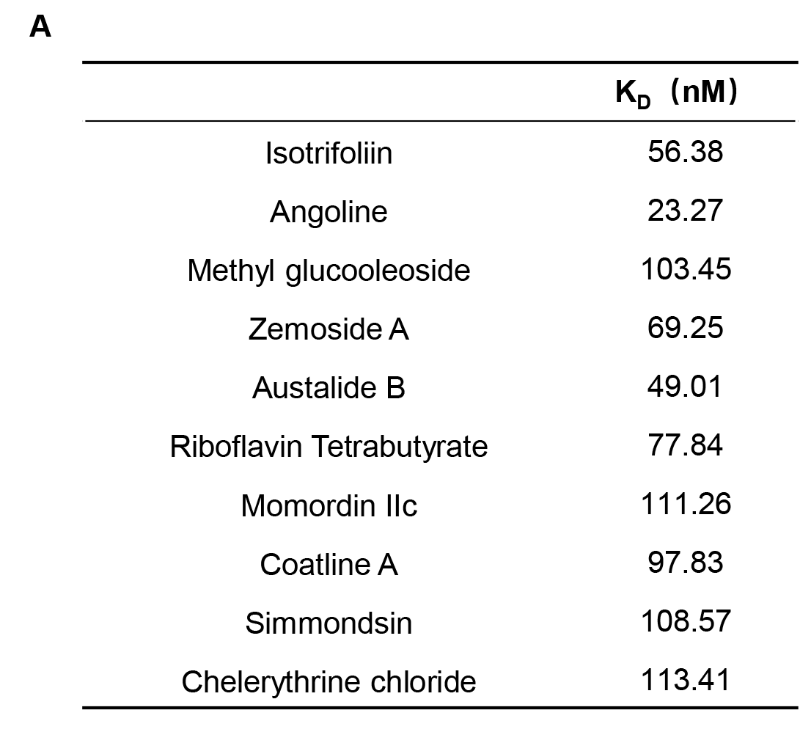


Fig S8. Affinity constant between MORC2 protein and different small molecule compounds.


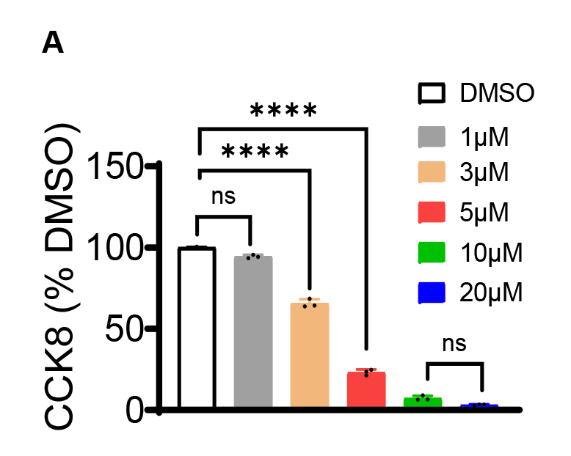


Fig S9. (A) The killing effect of Angoline on 4T1 cells based on different drug concentration.


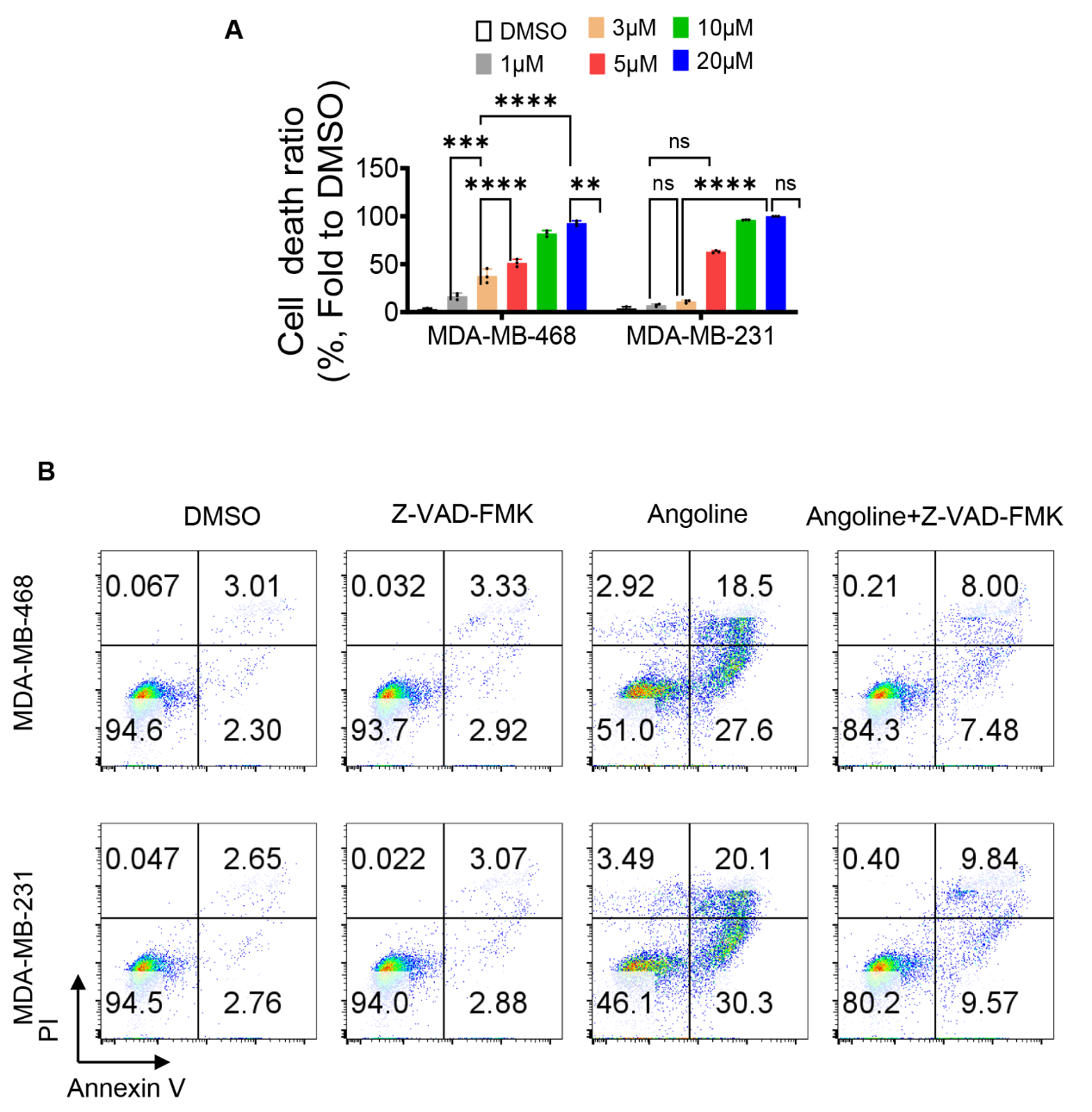


Fig S10. (A) Trypan blue staining results of TNBC cell death ratio after treatment with different concentrations of Angoline. (B) The incidence of apoptosis in TNBC cells treated with different drugs. ** P < 0.01; *** P < 0.001; **** P < 0.0001; ns, no significance.


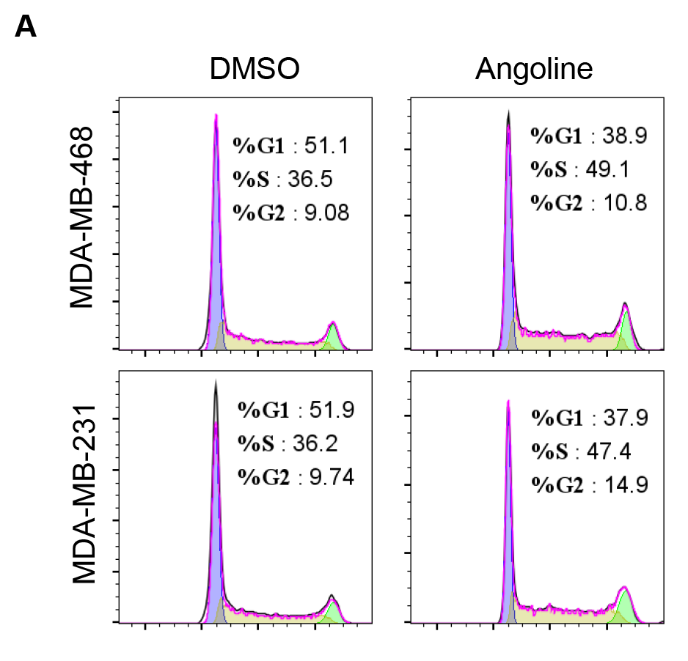


Fig S11. (A) Changes of cell cycle distribution in TNBC cells treated with Angoline.


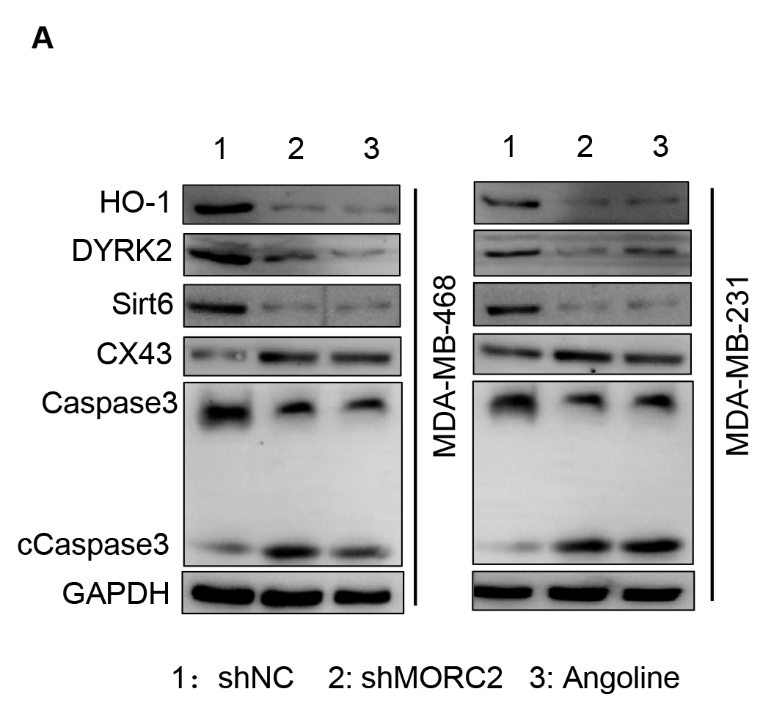


Fig S12. (A) Changes of cell cycle and apoptosis-related protein expression in TNBC cells after MORC2 knockdown or treated with Angoline.


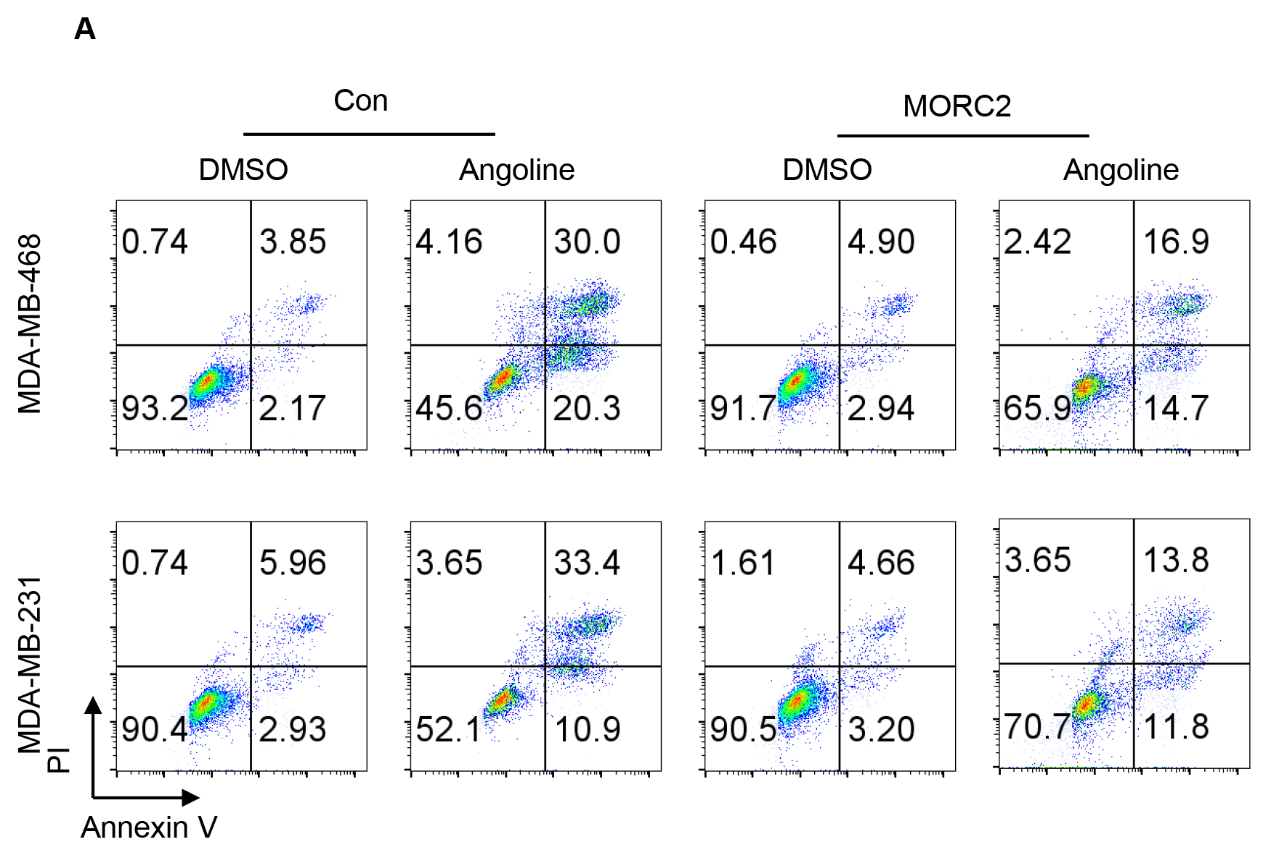


Fig S13. (A) Flow cytometry results of apoptosis changes in TNBC cells after MORC2 overexpressed and/or treated with Angoline.


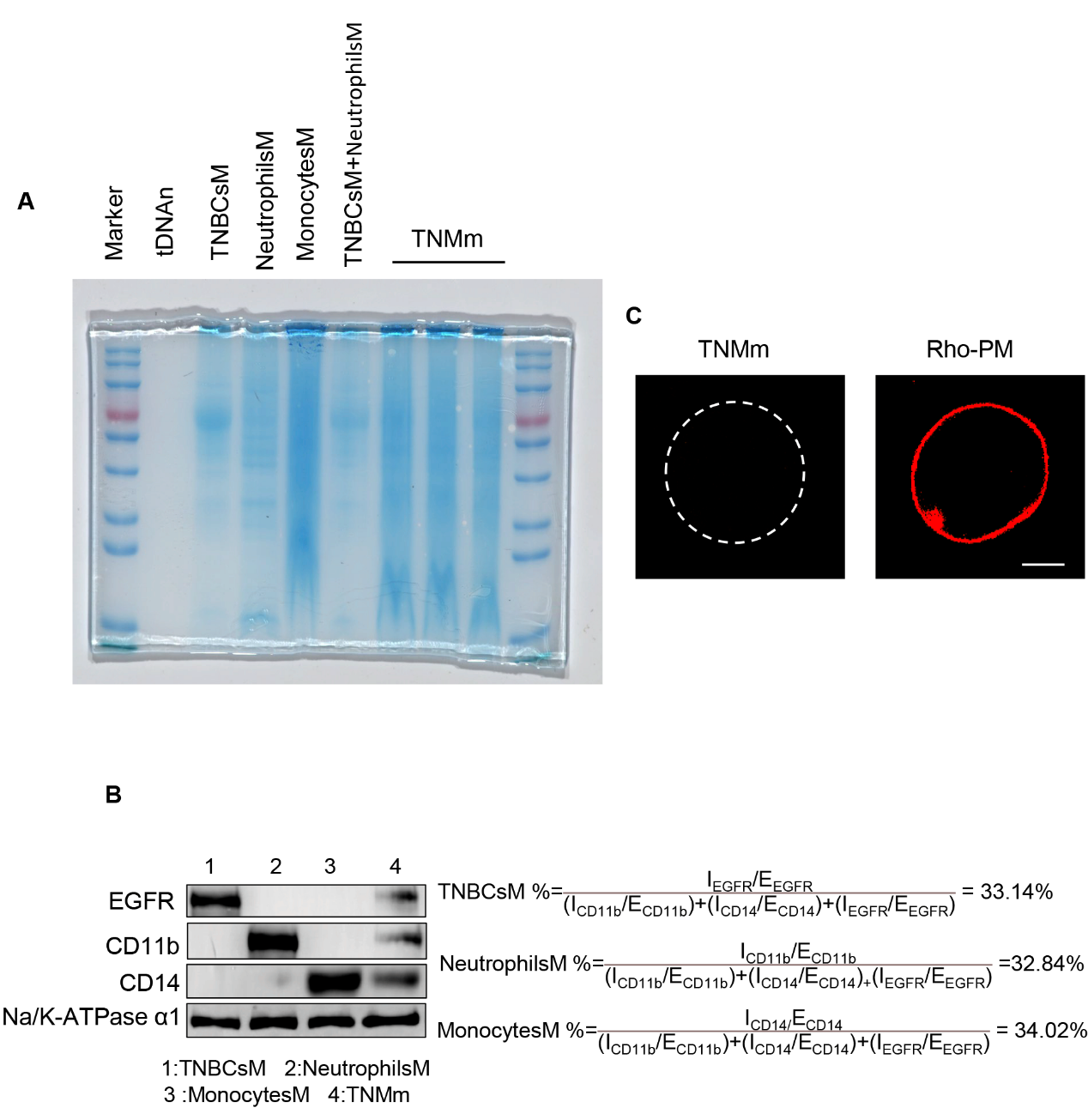


Fig S14. (A) SDS-PAGE protein analysis of markers, TNBC cells membrane, Neutrophils membrane, Monocytes membrane, tDNAn and TNMm. (B) The composition ratios of TNMm were calculated based on the Western blot results of specific membrane proteins from the membranes of TNBC cells, neutrophils, monocytes, and TNMm. I: Grayscale value of the TNMm band; E: Grayscale value of membrane brand from TNBC cells, neutrophils, or monocytes. (C) The fluorescent scan confirmed that the membrane of PM was successfully labeled with Rho.


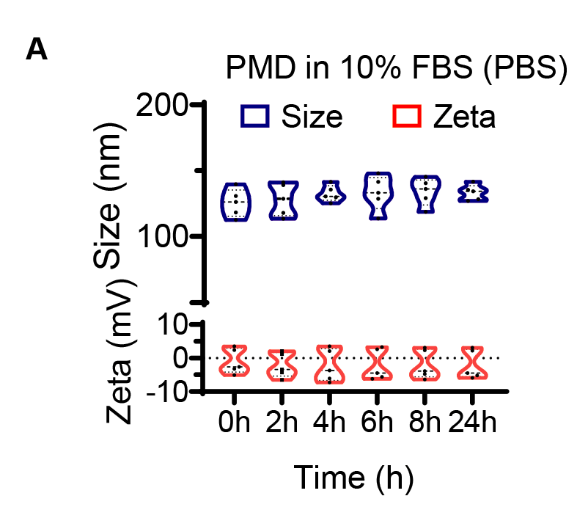


Fig. 15 (A) Stability of PMD in PBS containing 10% fetal bovine serum (FBS), which includes the average particle size and zeta potential change.


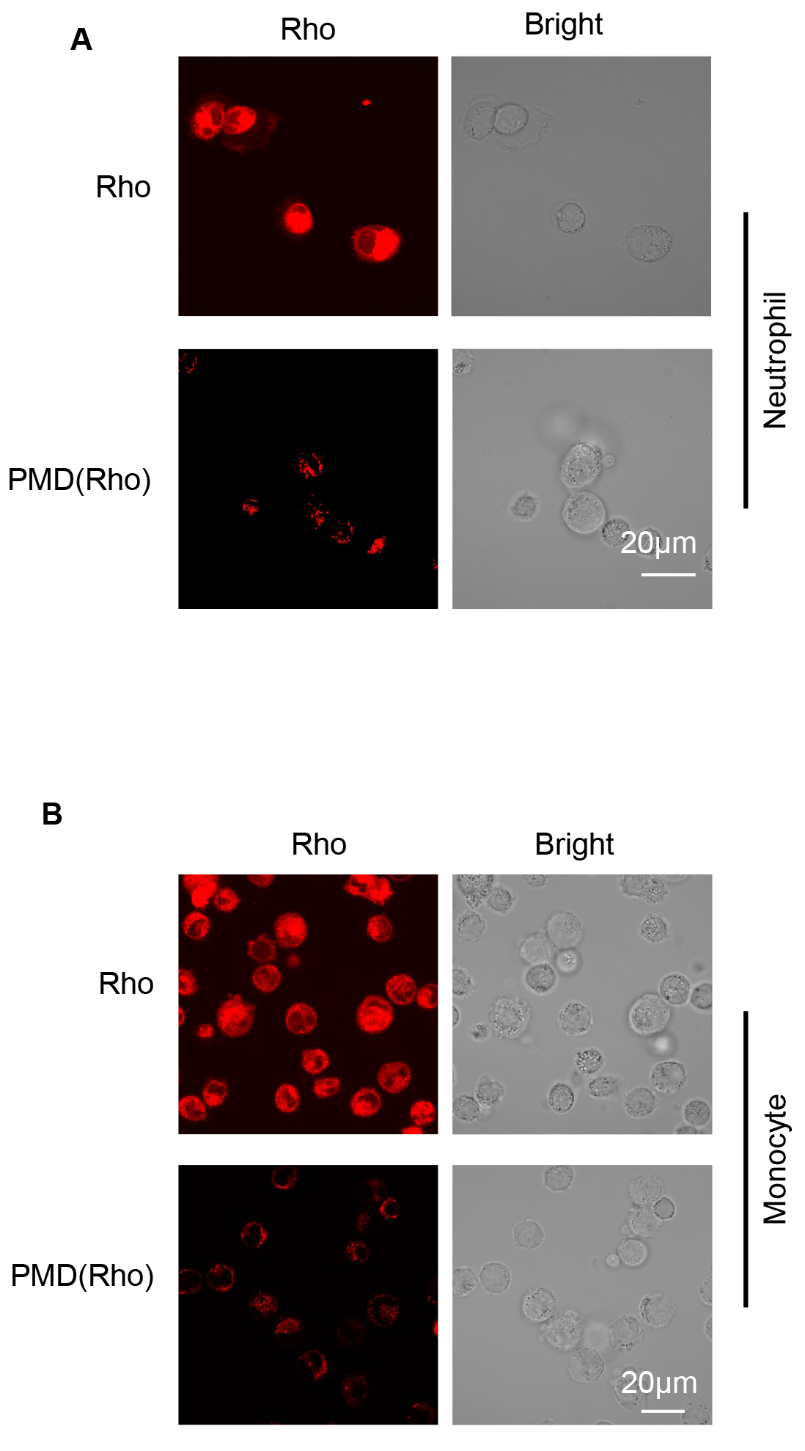


Fig S16. Fluorescence microscopy shows the phagocytosis of PMD(Rho) or Rho by neutrophils (A) and monocytes (B).


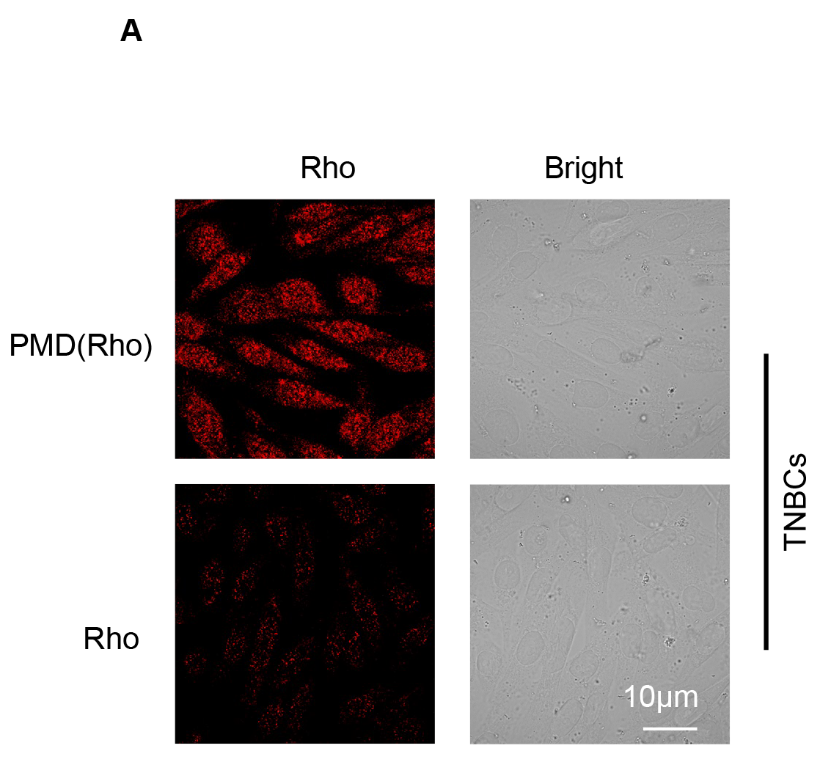


Fig S17. (A) Fluorescence microscopy shows the uptake of PMD(Rho) or Rho by TNBC cells.


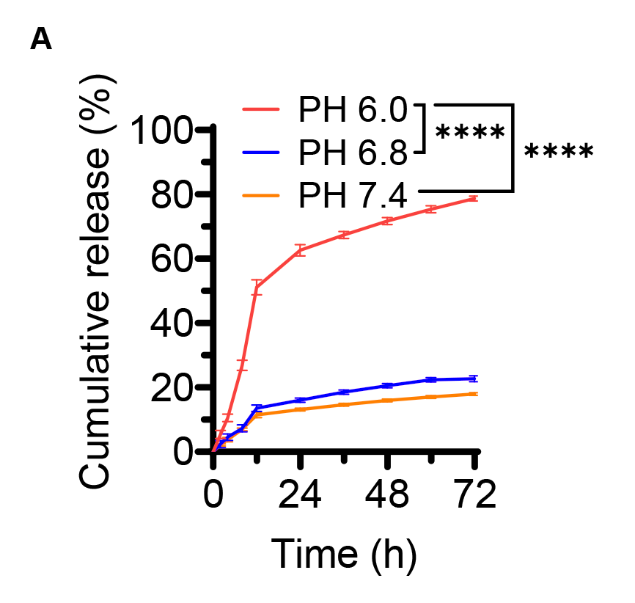


Fig. 18 (A) In vitro Angoline release from PMD (Angoline) in PBS (pH 6.0, 6.8, and 7.4) (n = 3).


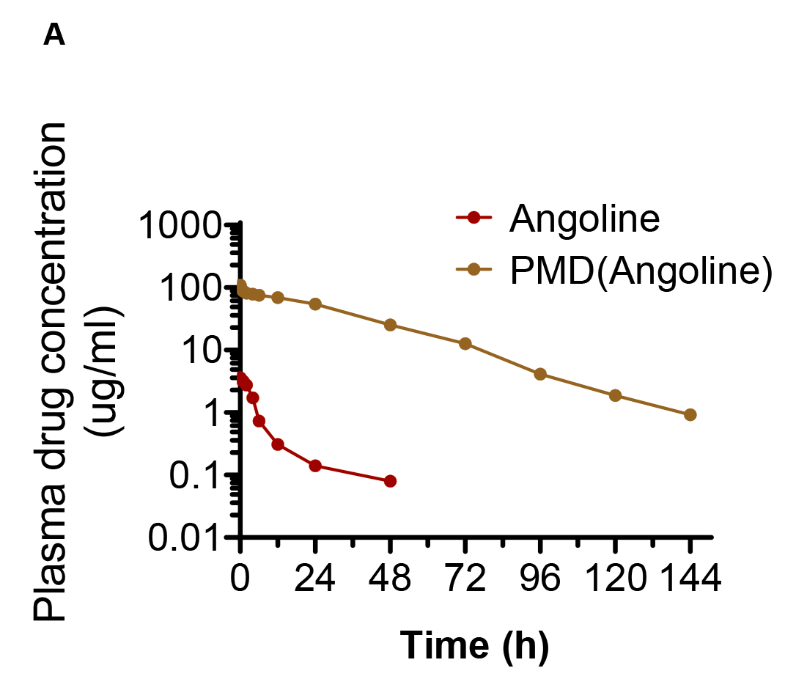


Fig. S19 (A) Plasma concentration-time profiles of Angoline in SD rats following a single intravenous injection of Angoline or PMD (Angoline) via the tail vein. Plasma Angoline levels were undetectable in SD rats 48 hours after administration of Angoline.


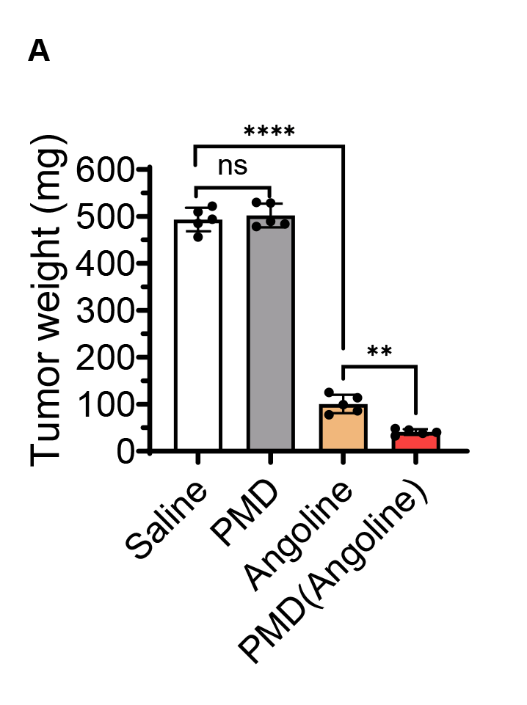


Fig. 20 (A) The weight of inoculated xenograft tumors treated with different drugs.
